# Spin Injection and Emission Helicity Switching in a 2D Perovskite/WSe_2_ Heterostructure

**DOI:** 10.1021/acs.nanolett.5c06501

**Published:** 2026-04-03

**Authors:** Jakub Jasiński, Francesco Gucci, Thomas Brumme, Swaroop Palai, Armando Genco, Alessandro Baserga, Jonas D. Ziegler, Takashi Taniguchi, Kenji Watanabe, Mateusz Dyksik, Christoph Gadermaier, Michał Baranowski, Duncan K. Maude, Alexey Chernikov, Giulio Cerullo, Agnieszka Kuc, Stefano Dal Conte, Paulina Plochocka, Alessandro Surrente

**Affiliations:** 1 Department of Experimental Physics, 414290 Faculty of Fundamental Problems of Technology, Wroclaw University of Science and Technology, 50-370 Wroclaw, Poland; 2 Department of Physics, 18981 Politecnico di Milano, Piazza Leonardo da Vinci 32, 20133 Milan, Italy; 3 Chair of Theoretical Chemistry, Technische Universität Dresden, Bergstraße 66, 01069 Dresden, Germany; 4 Laboratoire National des Champs Magnétiques Intenses, EMFL, CNRS UPR 3228, Université Grenoble Alpes, 38042 Grenoble, France; 5 Institute of Applied Physics and Würzburg-Dresden Cluster of Excellence ct.qmat, 9169 TUD Dresden University of Technology, 01062 Dresden, Germany; 6 Research Center for Materials Nanoarchitectonics, National Institute for Materials Science, 1-1 Namiki, Tsukuba 305-0044, Japan; 7 Research Center for Electronic and Optical Materials, National Institute for Materials Science, 1-1 Namiki, Tsukuba 305-0044, Japan; 8 Helmholtz-Zentrum Dresden-Rossendorf, HZDR, Bautzner Landstraße 400, 01328 Dresden, Germany; 9 Center for Advanced Systems Understanding, CASUS, Conrad-Schiedt-Straße 20, 02826 Görlitz, Germany; 10 Laboratoire National des Champs Magnétiques Intenses, EMFL, CNRS UPR 3228, Université Toulouse, Université de Toulouse 3, INSA-T, 31400 Toulouse, France

**Keywords:** 2D perovskite-transition metal dichalcogenide heterostructures, interlayer exciton, spin polarization, charge transfer

## Abstract

The ability to initialize and control long-lived spin populations in lead halide perovskites is essential to enable their use in spintronics technologies. Here, we show that the interlayer exciton observed in the photoluminescence spectrum of a (BA)_2_PbI_4_/WSe_2_ monolayer heterostructure below the intralayer exciton transitions exhibits circular dichroism, whose sign can be modulated by tuning the excitation energy. Equilibrium and transient absorption measurements reveal an additional absorptive feature between the A and B excitons of WSe_2_ with hybrid character. This resonance arises from hybridized valence band states of the two materials and plays a key role in controlling the helicity of the interlayer exciton emission. The tunable spin polarization demonstrated here, with the WSe_2_ monolayer effectively acting as a tunable spin filter, represents an important step toward the use of 2D perovskites in opto-spintronics applications.

## Graphical abstract

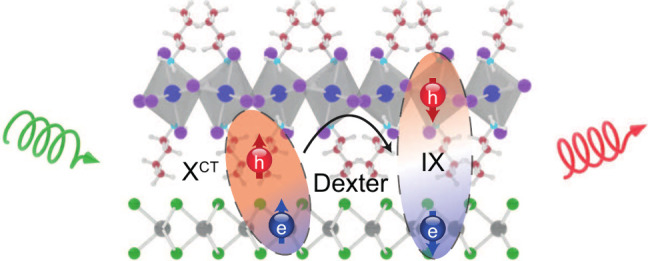

The use of a binary degree of freedom, such as carrier spin, to transport, store, and process information is the basic working principle of spintronic devices.^1^ A prerequisite for a material to possess spintronic functionalities^2^ suitable for optoelectronics^3,4^ is the ability to initialize a long-lived population of spin-polarized charge carriers. Lead halide perovskites exhibit a spin-polarized band structure,^5–8^ which is intrinsically related to their strong spin–orbit coupling,^9^ and helicity-dependent optical selection rules.^10–16^ These features make them excellent candidates for applications in opto-spintronics.^17^ While a large spin–orbit coupling is essential to efficiently initialize the spin polarization, it also leads to very short spin lifetimes, usually of the order of a few picoseconds.^11,15,18–20^

Multiple attempts have been made to overcome this limitation by tailoring structural parameters^11,16^ or electronic structure,^20^ exploiting polaronic effects,^21^ incorporating chiral organic spacers in two-dimensional (2D) lead halide perovskites,^13,22–27^ or decoupling the generation of spin-polarized carriers in moieties with chiral character and their radiative recombination in materials with a high optical quality.^12,28–31^ However, these efforts have often resulted in a relatively low degree of circular polarization of the emitted photoluminescence (PL),^12,28–30^ fundamentally limited by the strong spin–orbit coupling present in these materials.^32^ This strongly motivates the exploration of alternative approaches in which spin injection can be deliberately tailored to specific applications.

A class of materials that naturally lend themselves to acting as spin filters is TMD monolayers, due to their chiral optical selection rules.^33^ TMD/2D perovskite heterostructures^34,35^ have been extensively investigated for applications in high-efficiency photodetectors^36–39^ and for the study of charge and energy transfer.^14,40–46^ However, their use in the generation and transfer of spin-polarized carriers has remained limited,^14,41,47^ often relying on the use of 2D perovskites with chiral organic spacers,^41,47^ which are known to reduce the PL quantum yield.^13,48,49^ Moreover, this approach suffers from reduced flexibility since the spin of the carriers is fixed by the choice of the enantiomer used in the synthesis of the 2D perovskites. An alternative approach to the generation and transfer of spin polarized carriers in TMD/2D perovskite heterostructures is based on the combination of chiral optical selection rules of the TMD monolayer^33^ and type II band alignment.^42,43^ This enables efficient photoexcitation of spin-polarized carriers with a prolonged spin lifetime.^50^ Additionally, this approach offers improved control over the sign of the spin polarization, which is determined purely optically via the helicity of the excitation light. These factors help overcome the limitations inherent to lead halide perovskites andmake TMD/2D perovskite heterostructures highly promising for optospintronics.

Here, we exploit the chiral optical selection rules of WSe_2_ monolayers^33^ to inject a population of carriers with an externally controllable spin polarization into a (BA)_2_PbI_4_/WSe_2_ monolayer heterostructure. This leads to a circularly polarized PL of the interlayer exciton (IX). We achieve control over the sign of the PL circular polarization of the IX by tuning the excitation laser energy. Strikingly, we observe counter-polarized emission when the excitation laser is tuned in resonance with a heterostructure-related absorption feature, attributed to an interlayer charge transfer exciton (X^CT^) transition at higher energies. While this feature has been recently ascribed to hot electron transfer,^45^ we reveal its interlayer excitonic nature using a combination of density functional theory (DFT), helicity-resolved pump–probe spectroscopy, and linear absorption spectroscopy. Our results demonstrate a highly tunable approach for injecting spin-filtered carriers into a 2D perovskite and achieving all-optical control over the helicity of the emitted PL.

The heterostructure used in our investigation was fabricated by vertically stacking a (BA)_2_PbI_4_ flake and a WSe_2_ monolayer and is fully encapsulated in hBN to improve optical quality^51^ and minimize degradation under illumination or exposure to ambient conditions.^52,53^ A micrograph of the sample is shown in Figure S1­(a) of the Supporting Information. The micro-PL (μPL) and reflectivity spectra of the individual materials are discussed in Figure S3. In the heterostructure, the PL peaks related to the recombination of the intralayer exciton species of WSe_2_ are strongly quenched, as shown in Figure 1(a) (see also the PL map in Figure S1­(c)), due to charge and energy transfer across the heterostructure. The PL spectrum displays an additional broad peak at ≈ 1.55 eV, which is only observed in the heterostructure region, as confirmed by the PL map in Figure S1­(b). The energy of this peak shows good spatial uniformity, as seen in the map of Figure S2­(a). Consistent with previous work on this heterostructure,^14,41,47,54–57^ we attribute this feature to the recombination of an IX, with the electron and hole spatially separated in the TMD monolayer and the lead-halide octahedral plane, respectively, enabled by the type II band alignment.^14,40,41,43^ The band structure we calculated using DFT confirms this band alignment, as shown in Figure S4. Generally, the contribution of the organic spacers to the projected density of states of the valence band edge is not considered.^58,59^ However, our analysis demonstrates that the organic spacers provide a small, but non-negligible contribution to the valence band edge (see Figure S4).^42,43^ This leads to the cascaded band alignment in the valence band, shown schematically in the left panel of Figure 1(c). The possible presence of defects at the interface is not expected to change the type II band alignment,^60,61^ which enables the formation of the IX across the heterostructure. Moreover, the energy of the valence band edge of the various 2D perovskites is determined by the PbI_4_ framework. Consequently, the energy difference between the conduction band of WSe_2_ and the valence band of the 2D perovskites is essentially independent of the organic spacer.^43,55^ The energy of the IX PL of 2D perovskite/WSe_2_ heterostructures is consistently centered at ≈ 1.55 eV regardless of the specifics of the spacer,^55^ which confirms our assignment. The linear power dependence of the PL intensity, the lack of its saturation at high excitation, the power-induced blue shift of the IX PL peak (see Figure S5), and the temperature dependence of its energy and intensity (Figure S6) unambiguously demonstrate its interlayer nature.^41,47,54–57^ The slow dynamics exhibited by time-resolved PL of the IX shown in Figure S7 further confirm our assignment.^54–57^ We rule out energy transfer (as discussed in Figure S8),^41^ defect-related emission (see PL spectrum over a wide spectral range in Figure S9), self-trapping or strain as the possible origins of IX and X^CT^ in Sec. VIII of the Supporting Information. In the reflectivity spectrum of the heterostructure, shown in Figure 1(b), we identify the excitonic resonances of the individual materials and an additional resonance at ≈2.1 eV. We attribute this feature to an interlayer hybridized charge transfer exciton X^CT^ (see discussion of DFT calculations for more details), in which the hole occupies a hybridized state involving primarily the organic spacer and the TMD, as shown schematically in the right panel of Figure 1(c). In the reflectivity contrast spectrum, there are no resonances associated with the IX, which reflects its low oscillator strength.^62,63^

**Figure 1:**
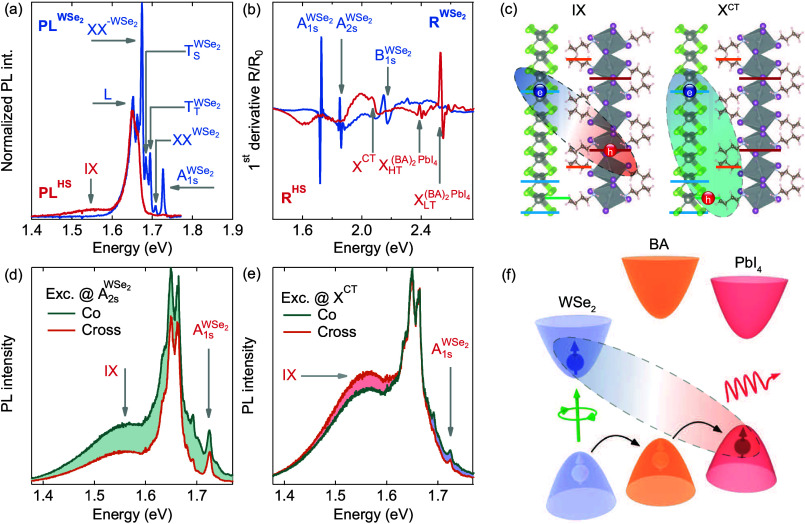
(a) PL and (b) reflectivity spectrum of the WSe_2_ monolayer and the heterostructure. A_1s_^WSe_2_^ indicates the 1s state of neutral exciton of WSe_2_, A_2s_^WSe_2_^ the 2s state, B_1s_^WSe_2_^ the B exciton, XX^WSe_2_^ the biexciton, T_T_^WSe_2_^ and T_S_^WSe_2_^ the triplet and singlet charged excitons, respectively, XX^–WSe_2_^ the charged biexciton, and L the localized excitons. X^CT^ designates the charge transfer interlayer exciton, and X_LT_^(BA)_2_PbI_4_^ and X_HT_^(BA)_2_PbI_4_^ the exciton of the low and high temperature phases of (BA)_2_PbI_4_, respectively. (c) Ball and stick model of the WSe_2_ monolayer/(BA)_2_PbI_4_ heterostructure with a schematic depiction of IX and X^CT^. The horizontal lines indicate the relative positions of the band edges. Helicity-resolved PL spectrum of the WSe_2_ monolayer/(BA)_2_PbI_4_ heterostructure for excitation in resonance with (d) the 2s exciton of WSe_2_ (A_2s_^WSe_2_^) and (e) the X^CT^ transition. The excitonic resonances are indicated by arrows. (f) Schematic band alignment of the TMD-perovskite heterostructure. Spin-conserving charge transfer of the hole from WSe_2_ to PbI_4_ and the formation of an interlayer exciton are schematically shown.

To demonstrate control over the circular polarization of the IX emission, we performed helicity-resolved PL measurements. We initially excite with a circularly polarized laser tuned in resonance with the 2s exciton of WSe_2_. The IX emission displays a strong cocircular polarization, inherited from the WSe_2_ intralayer exciton,^64^ as shown in Figure 1(d). The degree of circular polarization *P*_c_, defined as *P*_c_ = (*I*_co_ – *I*_cr_)/(*I*_co_ + *I*_cr_), where *I*_co/cr_ denotes the intensity of the copolarized and cross-polarized emission, respectively, amounts to ≈32% for the IX and to ≈26% for the WSe_2_ intralayer exciton. For intralayer excitons, copolarized PL arises from the spin–valley locking exhibited by TMD monolayers.^33^ Localized excitons exhibit a lower degree of circular polarization, in line with expectations.^65^ In the case of the IX, the copolarized PL originates from the interlayer transfer of spin-polarized holes from the valence band of the TMD monolayer to that of the 2D perovskite. This leads to the generation of a population of spin-polarized resident electrons in the conduction band of the TMD, which exhibit a significantly longer spin lifetime than that of intralayer excitons.^66,67^ The positive degree of circular polarization of IX arises from the recombination of these resident electrons with spin-polarized holes, whose transfer is demonstrated by helicity-resolved pump-probe measurements presented below. After the holes transfer to the 2D perovskite, the IXs inherit the spin-polarization induced by the excitation laser. Their recombination results in copolarized PL^14^ (see schematic in Figure 1(f)).

Intriguingly, we can invert the sign of the degree of circular polarization of the IX emission by tuning the excitation energy of the laser. The polarization-resolved PL spectrum of the heterostructure excited in the proximity of the B exciton of the WSe_2_ monolayer is shown in Figure 1(e). For this excitation condition, the degree of circular polarization of the WSe_2_ monolayer PL is negligible or slightly negative, due to a Dexter-like mechanism which couples same-spin states of K and K’ valleys.^64^ Unexpectedly, the IX PL exhibits opposite helicity with respect to the excitation laser. This effect is maximized when the laser is tuned to a resonance with X^CT^.

We measure the degree of polarization of the PL spectrum as a function of the photon energy of the excitation laser in the spectral region corresponding to the reflectivity spectrum in Figure 2(a). The PL intensity of WSe_2_ has a maximum for excitation energies corresponding to the B exciton of the WSe_2_ monolayer, as shown in Figure 2(b), while its degree of polarization exhibits a minimum at this resonance.^64^ Subsequently, we consider the helicity-resolved PL spectrum of IX as a function of the excitation energy. Its intensity, displayed in Figure 2(b), reaches its maximum over a relatively wide energy range, which includes both the intralayer B exciton and the interlayer X^CT^. From the data shown in Figure 2(b), we estimate the corresponding degree of circular polarization, reported in Figure 2(c) as a function of the excitation energy. Crucially, when we excite the heterostructure close to the resonance of X^CT^, the degree of circular polarization of the IX emission becomes increasingly negative, reaching ≈−10*%*. In contrast, in this spectral region, the degree of circular polarization of the WSe_2_ intralayer exciton increases with decreasing excitation energy. Otherwise, the trend of the degree of polarization follows that of the WSe_2_ intralayer exciton, which stems from charge transfer between the WSe_2_ monolayer and (BA)_2_PbI_4_.

**Figure 2:**
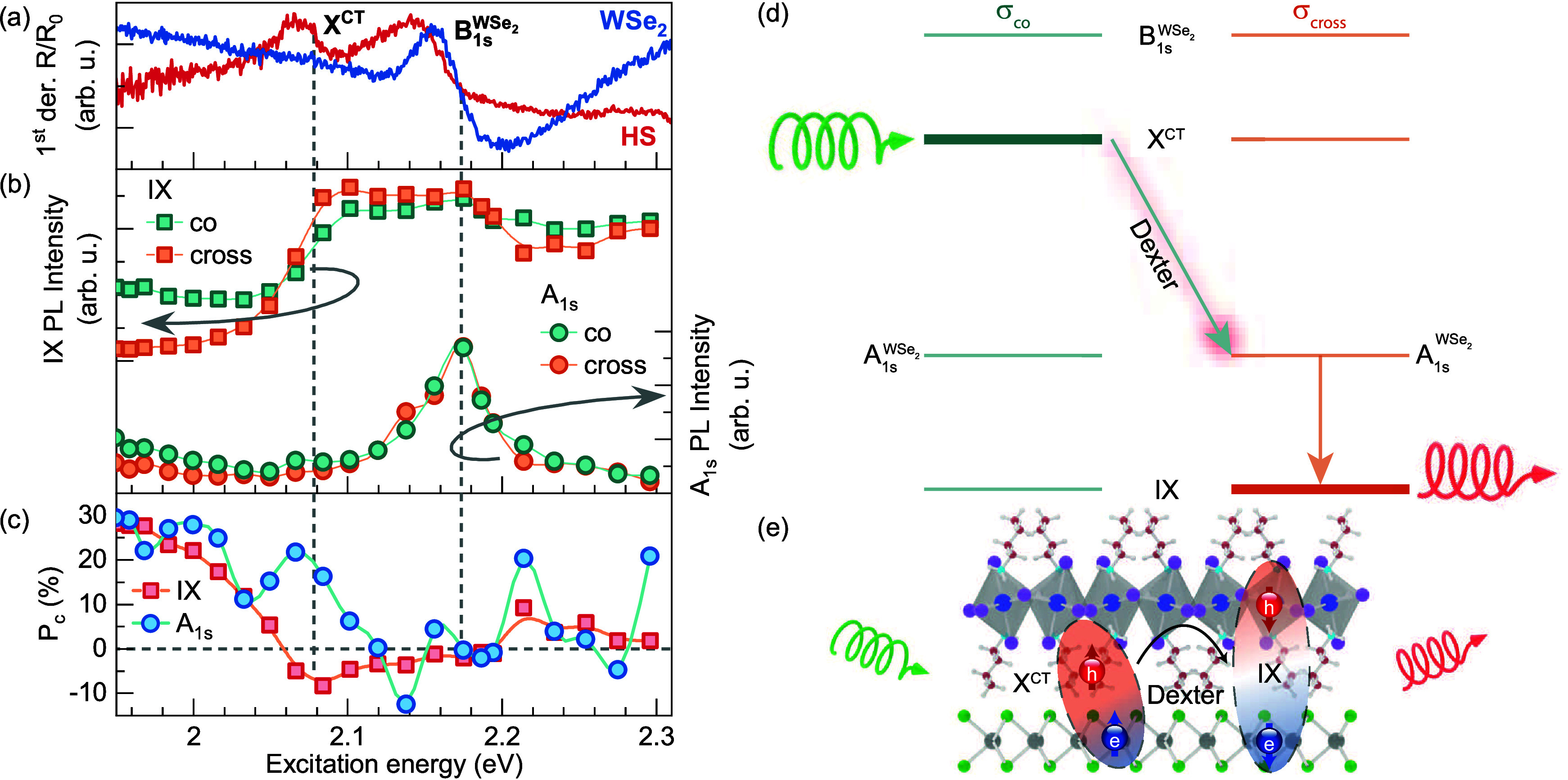
(a) First derivative of the reflectivity contrast spectrum measured on the WSe_2_ monolayer and on the heterostructure. (b) Helicity resolved PL intensity of the interlayer exciton and of the 1s exciton of the WSe_2_ monolayer and (c) degree of circular polarization *P*_c_ as a function of the excitation energy. The vertical dashed lines indicate excitonic resonances. (d) Schematic of transfer of the exciton population toward states not driven optically but with the same spin configuration as the optically driven state mediated by Dexter-like coupling. (e) Pictorial view of the reversed circular polarization emission of the interlayer exciton.

For excitation close to resonance with the B exciton, the degree of circular polarization of the WSe_2_ monolayer is determined by a Dexter-like coupling between exciton states with the same spin configuration.^64,68^ A similar process could explain the negative polarization of the IX. As discussed below, X^CT^ is likely to be formed from electron states at the K point originating from the conduction band of WSe_2_, while hole states are mainly formed from those of the organic spacer of the 2D perovskite at the K point, which hybridize with states from the spin–orbit split valence band of WSe_2_. This yields an interlayer exciton complex with a partial intralayer character, similar to interlayer excitons in bilayer TMDs.^69^ Thus, the negative polarization of IX could be explained if the interlayer X^CT^ inherits the same spin configuration of the WSe_2_ B exciton following this hybridization. In this case, the resonant excitation of X^CT^ would enable an efficient transfer of spin-polarized excitons, a process similar to the Dexter-like coupling between B and A excitons of opposite valleys in TMD monolayers.^68^ The exciton population is transferred from the valley where it is optically initialized to the A exciton state of WSe_2_ with the same spin orientation and belonging to the valley that is not optically addressed. This valley couples to the circular polarization with opposite helicity, with respect to the excitation laser. From the A exciton of WSe_2_, the excitons can relax to populate the IX, which will then preferentially emit photons with the opposite helicity relative to the excitation laser, as schematically shown in Figure 2(d). This process leads to the emission of counter-polarized PL from the IX, as illustrated in Figure 2(e). The presence of electronic coupling and charge transfer between the (BA)_4_PbI_4_ and the WSe_2_ monolayer is therefore pivotal in sensitizing the IX with the optical selection rules of WSe_2_, which enables a fully optical control over the spin transferred from WSe_2_ to (BA)_2_PbI_4_ and the helicity of the IX emission.

To understand the origin of X^CT^ and identify possible direct interlayer charge transfer transitions at energies between those of the A and B excitons of WSe_2_, we calculated the electronic structure of the interface (see Figure S4). The calculation reveals the presence of states that originate from the 2D perovskite at the K point, which are energetically located between the spin–orbit split valence bands of the WSe_2_ monolayer, labeled VB1 and VB2 in Figure 3(a). For the region around the K point, we calculated the spin expectation values, σ_*z*_, to reveal the spin components in the *z* direction (perpendicular to the layers), as shown in Figure 3(a). In Figure 3(b), we plot the isosurface of one of the eigenstates found between the spin–orbit split valence bands of the WSe_2_ monolayer, labeled Hyb in Figure 3(a). Although this state originates from the (BA)_2_PbI_4_, the isosurface demonstrates that in the heterostructure it exhibits some hybridization with d-orbital-like states of the transition metal atom in the WSe_2_ monolayer.^70^ The Hyb state at 75 meV above the valence band maximum could be responsible for the interlayer X^CT^ resonance. This energy difference is compatible with the ≈80 meV shift of the X^CT^ transition observed in the differential reflectivity spectrum of the heterostructure with respect to the B exciton of the WSe_2_ monolayer (see Figure 2(a)). The observation of this state in reflectivity measurements suggests that X^CT^ has a non-negligible oscillator strength, which can be attributed to its partial intralayer character due to the hybridization shown in Figure 3(b). Moreover, the spin polarization of the hybridized states supports the role of Dexter-like coupling between the same-spin states as the mechanism behind the observed negative circular polarization of the IX PL, which stems from the radiative recombination of electrons confined in the TMD monolayer and holes in the lead halide slab of the 2D perovskite.

**Figure 3:**
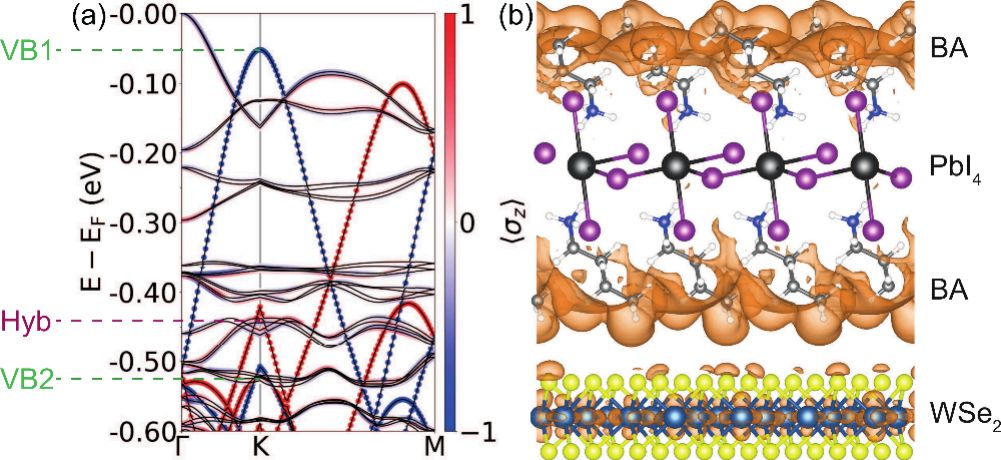
(a) Zoom-in to the valence band of the (BA)_2_PbI_4_/WSe_2_ heterostructure from Figure S4 together with the spin expectation values, σ_*z*_. Bands plotted with full dots originate from the WSe_2_ monolayer, while states plotted with a colored shading originate from (BA)_2_PbI_4_. VB1 and VB2 indicate the spin–orbit split valence bands of WSe_2_, while Hyb designates the hybridized states involved in the X^CT^ transition. (b) Eigenstate of one of the hybridized states involved in the X^CT^ transition. An isosurface value of 1× 10^–6^ Å^–3^, which is the probability density of this state, was used. This corresponds to about 2.8 × 10^–8^ electron/Å^3^. For comparison, for the valence band maximum of WSe_2_, these values are 1 × 10^–3^ Å^–3^ and 2.8 × 10^–5^ electron/Å^3^, respectively.

To investigate the dynamics of charge and spin transfer and exciton dynamics in the heterostructure, we performed broadband optical pump–probe spectroscopy (see the schematic in Figure 4(a)). We initially tuned the energy of the pump beam above the optical bandgap of both constituents of the heterostructure. The complete transient reflectivity maps of the WSe_2_ monolayer and of the heterostructure as a function of the pump–probe delay and the probe energy are shown in Figure S10. From these maps, we extract the transient reflectivity spectra Δ*R*/*R* (Δ*R* is the transient variation of reflectivity after pump excitation, while *R* indicates the static reflectivity) at a fixed delay, which we show in Figure 4(b). The transient reflectivity spectrum of the WSe_2_ monolayer is characterized by a transient signal at the energies of the 1s and 2s states of the A exciton as well as the B exciton. The transient reflectivity spectrum of the heterostructure displays all excitonic resonances of WSe_2_, broadened due to the presence of additional nonradiative recombination channels in the heterostructure,^71^ together with an additional transient signal at 2.08 eV, assigned to the X^CT^ resonance (see Figure 4(b)). We attribute the transient signals measured at the energies of the A and B exciton resonances of WSe_2_ under nonresonant photoexcitation primarily to photobleaching and broadening of the excitonic peaks.^72^ The energy renormalization of the excitonic peaks is almost negligible under these excitation conditions.^72^ The positive transient signal at the energy of X^CT^ is attributed to a Pauli blocking process.^73^ Coulomb screening induced by electron–hole pairs generated after pump excitation would result in an energy shift of the X^CT^ peak and consequently in a derivative-like transient signal. We show the temporal dynamics of the X^CT^ resonance in Figure 4(c). This signal exhibits an instantaneous rise time, similar to the bleaching dynamics of the B exciton resonance of WSe_2_, also displayed in Figure 4(c), and is limited by the instrument response function of the setup. Based on the results of DFT calculations, a possible mechanism that leads to instantaneous formation of X^CT^ could be the direct excitation of an interlayer charge transfer transition, as already observed in WS_2_/graphene heterostructures.^73^ In this scenario, electrons from hybridized electronic states located between the spin–orbit split valence bands of the TMD are directly promoted by light excitation to the conduction band of WSe_2_.

**Figure 4:**
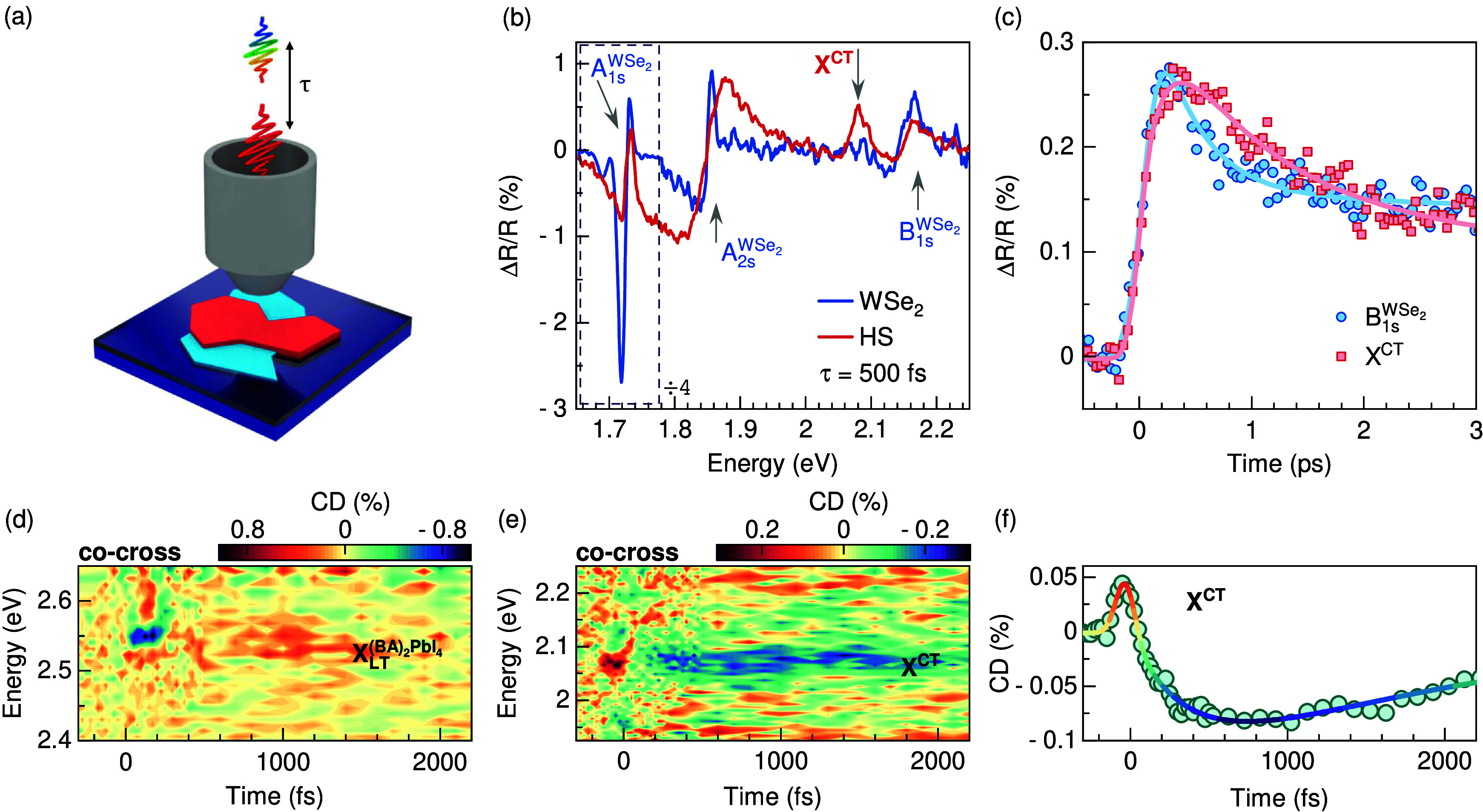
(a) Schematic of broadband pump–probe measurements performed on the (BA)_2_PbI_4_/WSe_2_ heterostructure. (b) Transient reflectivity spectrum of the heterostructure and of the isolated WSe_2_ monolayer excited at 2.64 eV (above the optical band gap of both materials) and extracted at a delay τ = 500 fs. The excitonic resonances are indicated. The resonance corresponding to the WSe_2_ A exciton (A_1s_^WSe_2_^) has been rescaled for increased clarity. (c) Transient differential reflectivity of the B exciton (B_1s_^WSe_2_^) and of the interlayer charge transfer exciton (X^CT^) measured as a function of the pump–probe delay. The line is the fit of an exponential rise and a biexponential decay model to the experimental data. Transient circular dichroism (CD = (Δ*R*/*R*)_co_ – (Δ*R*/*R*)_cr_) maps of the (d) exciton transition of the low temperature phase of (BA)_2_PbI_4_ (X_LT_^(BA)_2_PbI_4_^) and (e) X^CT^ excited in resonance with the WSe_2_ A exciton obtained by subtracting the cross-polarized transient absorption from the copolarized transient absorption. (f) Dynamics of the transient circular dichroism of X^CT^ as a function of the pump–probe delay. The line is the fit to an exponential rise and decay model.

Polarization-resolved pump–probe measurements provide access to transient spin-dependent dynamics. Using linearly polarized pump pulses resonant with the 1s exciton of WSe_2_, we observe a distinct bleaching signal at the energy of the exciton of (BA)_2_PbI_4_ as a consequence of an interlayer hole transfer process from the WSe_2_ layer to the 2D halide perovskite (see Figure S11). We investigate the interlayer transfer dynamics of spin-polarized carriers by performing helicity-resolved optical pump–probe measurements. Due to spin–valley locking in monolayer TMDs,^33^ we selectively photoexcite a population of spin-polarized electron–hole pairs in the TMD layer upon resonant excitation of the A exciton 1s transition of WSe_2_ with circularly polarized pump pulses. The temporal evolution of the spin-polarized carriers is measured using copolarized and cross-polarized broadband probe pulses. We show in Figure 4(d) the transient circular dichroism (CD) as a function of the probe photon energy in the spectral region of (BA)_2_PbI_4_ exciton and of the pump–probe delay. The CD is defined as the difference between the transient reflectivity signals co- and cross-polarized to the pump laser (CD = (Δ*R*/*R*)_co_ – (Δ*R*/*R*)_cr_) and is proportional to the spin polarization of the exciton population. The spectrally resolved transient response initially displays a sharp feature at zero delay, which can be attributed to a coherent effect related to the spin/valley-dependent optical Stark shift of excitonic resonances.^74^ At later delays, a weak but non-negligible positive transient signal develops at the energy of the (BA)_2_PbI_4_ exciton. This results from the photogeneration of spin-polarized holes only in WSe_2_ upon its resonant excitation, followed by their directional transfer to (BA)_2_PbI_4_, similar to previous observations in heterobilayers based on TMDs.^75^ The positive CD is in agreement with the degree of circular polarization of IX observed in PL measurements under similar excitation conditions. The hole transfer preserves the optically injected spin polarization. The radiative recombination of the resulting IX leads to the emission of a photon copolarized with respect to the excitation laser.

The transient CD in the spectral range of X^CT^ is shown in Figure 4(e). Crucially, we notice a pronounced negative signal at energies corresponding to X^CT^. By integrating the CD spectrum across this energy interval, we obtain the dynamics of its spin polarization,^76–78^ which we show in Figure 4(f). Also in this case, the early dynamics are dominated by a sharp positive feature at zero delay related to the spin/valley dependent optical Stark shift of excitonic resonances.^74^ The opposite sign of the CD is consistent with the reversed optical selection rules characteristic of X^CT^. A possible mechanism leading to this is related to the Dexter-like coupling between the WSe_2_ A exciton resonantly excited and the B exciton, from which the holes could relax to hybridized states that contribute to the X^CT^ resonance. The rise of the negative CD might reflect this relaxation process of the spin-polarized holes. The decay of the CD is mainly driven by the depolarization via electron–hole exchange interaction,^14,19,20,79^ which is expected to be relatively inefficient in this case, due to the reduced overlap of the electron–hole wave functions located in different layers.^50^

In conclusion, we have engineered a (BA)_2_PbI_4_/WSe_2_ monolayer heterostructure with the goal of controlling the helicity of circularly polarized light emitted from a 2D perovskite with the helicity tubable via the excitation energy. To reach this goal, we optically initialize the spin polarization of charge carriers in the WSe_2_ monolayer and exploit the spin-conserving charge transfer of holes toward (BA)_2_PbI_4_, which enables the emission of circularly polarized light via the recombination of the IX. In the differential reflectivity spectrum of the heterostructure, we observe a new excitonic resonance at a slightly lower energy than that of the B exciton of WSe_2_. When the circularly polarized excitation is resonant with this transition, the IX PL strikingly exhibits a circular polarization opposite that of the excitation laser. The calculated band structure demonstrates the emergence of spin-polarized hybrid states between the A and B excitons of WSe_2_, which participate in the formation of this interlayer X^CT^. Helicity-resolved pump–probe measurements demonstrate that X^CT^ exhibits a negative circular dichroism when the heterostructure is pumped in resonance with the A exciton of the WSe_2_ monolayer. In general, we show that the WSe_2_ monolayer can act as a spin filter to efficiently initialize a spin population in 2D perovskites, whose amount and sign can be conveniently controlled with a fully optical approach. These results can pave the way for the flexible design of optospintronic devices with an optically controllable circular dichroism.

## Supplementary Material



## Notes

Methods: sample fabrication, linear and pump–probe optical spectroscopy, band structure calculations; PL energy and intensity maps of intra- and interlayer excitons; detailed discussion and assignment of the PL and differential reflectivity peaks; note on band structure calculations; excitation power and temperature dependence of the PL spectrum of the heterostructure; time-resolved PL measurements of the interlayer exciton; discussion on possible alternative origins of the IX and X^CT^ peaks; pump-prove reflectivity maps and discussion on the dynamics of the charge transfer (PDF)
