# Supplementary material for: Spin Injection and Emission Helicity Switching in a 2D Perovskite/WSe2 Heterostructure
Source: Nano Lett. 2026 Apr 3;26(14):4621–9. doi: 10.1021/acs.nanolett.5c06501 (PMC13299003; doi:10.1021/acs.nanolett.5c06501)
Supplement: Supplementary file 1 [file nl5c06501_si_001.pdf]

Supplementary Information

Spin injection and emission helicity switching in a 2D perovskite/WSe_2 heterostructure

Jakub Jasiński,[1,2,*] Francesco Gucci,[3,*] Thomas Brumme,[4] Swaroop Palai,[2] Armando Genco,[3] Alessandro Baserga,[3] Jonas D. Ziegler,[5] Takashi Taniguchi,[6] Kenji Watanabe,[7] Mateusz Dyksik,[1] Christoph Gadermaier,[3] Michał Baranowski,[1] Duncan K. Maude,[2] Alexey Chernikov,[5] Giulio Cerullo,[3] Agnieszka Kuc,[8,9] Stefano Dal Conte,[3,†] Paulina Plochocka,[1,2,‡] and Alessandro Surrente[1,§]

[1]*Department of Experimental Physics, Faculty of Fundamental Problems of Technology, Wroclaw University of Science and Technology, 50-370 Wroclaw, Poland*

[2]*Laboratoire National des Champs Magnétiques Intenses, EMFL, CNRS UPR 3228, Université Grenoble Alpes, Université Toulouse, Université Toulouse 3, INSA-T, Grenoble and Toulouse, France*

[3]*Department of Physics, Politecnico di Milano, Piazza Leonardo da Vinci 32, 20133 Milan, Italy*

[4]*Chair of Theoretical Chemistry, Technische Universität Dresden, Bergstraße 66, 01069 Dresden, Germany*

[5]*Institute of Applied Physics and Würzburg-Dresden Cluster of Excellence ct.qmat, Technische Universität Dresden, 01062 Dresden, Germany*

[6]*International Center for Materials Nanoarchitectonics, National Institute for Materials Science, Tsukuba, Ibaraki 305-004, Japan*

[7]*Research Center for Functional Materials, National Institute for Materials Science, Tsukuba, Ibaraki 305-004, Japan*

[8]*Helmholtz-Zentrum Dresden-Rossendorf, HZDR, Bautzner Landstraße 400, 01328 Dresden, Germany*

[9]*Center for Advanced Systems Understanding, CASUS, Conrad-Schiedt-Straße 20, 02826 Görlitz, Germany*

(Dated: February 16, 2026)

I. METHODS

Fabrication and optical spectroscopy

The sample was fabricated using flakes mechanically exfoliated on PDMS and then assembled on the silicon substrate flake by flake via the dry transfer method [1]. Photoluminescence (PL) and reflectivity spectra have been obtained at cryogenic temperatures of $\sim 4\,\mathrm{K}$, unless otherwise specified. For PL and PL excitation (PLE) measurements, the sample was mounted on the cold finger of a He flow cryostat. A pulsed Ti-sapphire laser (80 MHz repetition rate, 150 fs pulse width) was used to pump an optical parametric oscillator, which was used as the excitation source in PLE measurements, to achieve wavelength tuning in a wide range. For these measurements, the laser was tuned below the exciton resonance of $(BA)_2PbI_4$ to avoid that the low energy tail of its PL would overcome the PL of WSe_2. This would make the extraction of the PL intensity of WSe_2 inaccurate. For polarization-resolved measurements, an achromatic quarter-wave plate was mounted immediately before the microscope objective, with the fast axis rotated by 45° with respect to the transmission axis of a polarizer. The excitation laser was converted to circular polarization, and the backscattered PL was converted again to linear polarization. The polarization state of the PL signal was analyzed by a combination of an achromatic half-wave plate and a linear polarizer. For excitation power and temperature-dependent PL measurements, 640 nm or 630 nm wavelengths were used. A 50× microscope objective with a numerical aperture 0.55 was used to focus the excitation laser on the sample and collect the signal with a spatial resolution of $\sim 1\,\mu\mathrm{m}$. The signal was directed to a spectrometer equipped with a liquid-nitrogen-cooled CCD camera. For reflectivity measurements, a white light source was used instead of the laser. Position dependent PL measurements were performed with a scanning step of 1 µm along both horizontal and vertical directions. The scans were enabled by using an automated xy translation stage on which the cryostat was mounted. For PL maps of the WSe_2 and the heterostructure region, a 532 nm CW laser was used. For PL maps of the 2D perovskite flake, the wavelength of the Ti-sapphire laser was tuned to 430 nm to achieve above band gap excitation conditions. Time-resolved PL decays were measured by using

* These authors contributed equally to the work
† stefano.dalconte@polimi.it
‡ paulina.plochocka@lncmi.cnrs.fr
§ alessandro.surrente@pwr.edu.pl

a Supercontinuum white light laser by NKT Photonics as the excitation source with the repetition rate set to 20 MHz. These measurements were performed at 80 K to maximize the signal. The broadband light pulse was spectrally filtered by a monochromator. The PL signal was spectrally filtered with a bandpass filter to select only the emission of the interlayer exciton. The spectrally filtered PL signal was fed into an avalanche photodiode. This detector was connected to a time-correlated single-photon counter module, which allowed us to reconstruct the time-resolved histogram of single-photon detection events.

Band structure calculation

The model of the $(BA)_2PbI_4/WSe_2$ heterostructure was created using a coincidence lattice algorithm implemented in hetbuilder [2] with less than 0.2% strain on the individual layers. The $(BA)_2PbI_4/WSe_2$ heterostructure in the low-temperature phase consists of 207 atoms and can be characterized by supercell vectors $m_1 = (-1, -4)$, $m_2 = (4, 7)$, $n_1 = (0, -1)$ and $n_2 = (1, 2)$ with a rotation angle of $\theta = 20.65°$ and a vacuum spacing of 100 Å, see Ref. [2] for details (other representation choices are possible). This model structure was relaxed such that the supercell matched the hexagonal symmetry of WSe_2. The difference from a fully relaxed lattice was below 0.1%. To fully optimize the heterostructure model, we used FHI-aims [3] employing the Perdew-Burke-Ernzerhof (PBE) functional [4] on tight tier 1 numeric atom-centered orbitals, including the nonlocal many-body dispersion correction (MBD-nl) [5, 6], and scalar relativistic corrections (ZORA) on a $6 \times 3 \times 1$ Γ-centered k-grid. The γ-angle was kept fixed, while forces and stresses were minimized until below $0.01\,\mathrm{eV\AA^{-1}}$. The SCF parameters were automatically adjusted after three steps. The resulting lattice vectors of the fully optimized system are $a = 8.759$ Å, $b = 18.433$ Å, and $\gamma = 91.94°$. The electronic band structure, the Mulliken projections, and the density of states were calculated including spin-orbit coupling (SOC) and considering the dipole correction on a $12 \times 6 \times 1$ Γ-centered k-grid.

Transient reflectivity measurements

The experimental setup used for the transient reflectivity measurements has been described in detail elsewhere [7]. It is based on an amplified Ti:sapphire laser, which generates

~ 100 fs pulses at 800 nm (1.55 eV) with a repetition rate of 2 kHz. The output of the laser is split into two beams (pump and probe). The pump pulses can be continuously tuned in energy between 470 nm (2.64 eV) to 780 nm (1.59 eV) with a bandwidth of ~ 10 nm, by a non-collinear optical parametric amplifier (NOPA). The probe pulses are focused onto a sapphire crystal to generate a broadband supercontinuum, whose spectrum can be appropriately filtered using band-pass filters. A mechanical chopper is used to modulate the pump beam at 250 Hz, while a mechanical delay line controls the pump-probe temporal delay. Both beams are circularly polarized by making use of a polarizer and an achromatic wave plate when appropriate and are collinearly focused onto the sample with an objective lens (8 mm focal length, numerical aperture 0.3). The diameter of the probe beam at the sample position is estimated to be ~ 3 µm, while the pump is slightly defocused to obtain a larger spot size. The sample is housed in a closed-cycle helium cryostat. All pump-probe measurements are performed at a base temperature of 8 K. The probe beam reflected from the sample is collected through the objective lens and directed to a dispersive spectrometer equipped with a CCD detector (Princeton Instruments PIXIS 100) triggered by the laser.

II. PL MAPS

In Fig. S1(a), we show a micrograph of the investigated sample. Mechanical exfoliation of a WSe_2 bulk crystal yields monolayer and bilayer areas, identified on the micrograph by a continuous and dashed contour, respectively. The heterostructure is finalized by transferring a mechanically exfoliated $(BA)_2PbI_4$ flake and encapsulating the structure in hBN.

To reveal the impact of the heterostructure on the PL spectrum, we measured PL maps. Fig. S1(b) summarizes the spatial dependence of the intensity of the low energy peak attributed to the interlayer exciton (IX). As confirmed by comparing the PL map with the micrograph of Fig. S1(a), this peak is only observed in areas where WSe_2 and $(BA)_2PbI_4$ overlap. This backs the assignment of the low energy peak in the PL spectrum of the heterostructure to the IX. In Fig. S1(c), we show the PL map obtained by integrating the peak corresponding to the intralayer WSe_2 exciton. The intensity of this signal is considerably decreased in areas corresponding to the heterostructure, as expected for a type II band alignment. The spatial dependence of the exciton peak related to the low-temperature phase of $(BA)_2PbI_4$ is depicted in Fig. S1(d). Although the 2D perovskite flake almost completely

overlaps with WSe_2, there is a thin part of it that is not in direct contact. However, the PL intensity of $(BA)_2PbI_4$ seems to be fairly unaffected by the presence of a different material and is mainly influenced by the local thickness of the flake. The weak dependence of the intensity of the 2D perovskite PL suggests that only emission from regions close to WSe_2 is affected by the charge transfer process, while the effect on the global flake scale is relatively small.

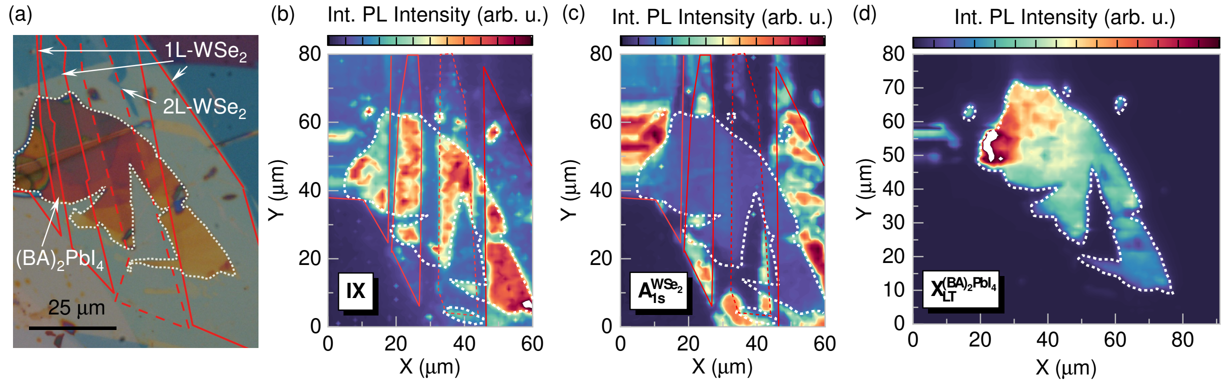

FIG. S1. (a) Micrograph of the investigated $(BA)_2PbI_4/WSe_2$ heterostructure. The contour of monolayer WSe_2 is highlighted by continuous red lines, that of bilayer WSe_2 by dashed red lines. The $(BA)_2PbI_4$ flake is traced in a dashed white line. PL intensity map of (b) the IX, (c) the intralayer WSe_2 exciton and (c) the intralayer exciton of the $(BA)_2PbI_4$ low temperature phase.

The maps of the PL energy of the IX, intralayer WSe_2 exciton and intralayer $(BA)_2PbI_4$ exciton are shown in Fig. S2(a,b,c), respectively. The PL energy of the IX is spatially uniform and varies between 1.55 eV to 1.56 eV. This is expected, because the PL energy of the IX is determined by the energy difference of the conduction band of WSe_2 and the valence band of $(BA)_2PbI_4$, with a limited influence of the surrounding and the dielectric environment between the layers [8]. A more significant influence of the dielectric environment could be expected on the excitonic resonance of the WSe_2 monolayer [9]. Unfortunately, this effect is masked by the complete suppression of the PL of the WSe_2 monolayer on the heterostructure, which prevents us from extracting reliably the PL energy of the intralayer exciton, as shown in Fig. S2(b). Finally, Fig. S2(c) demonstrates how the energy of the intralayer exciton of $(BA)_2PbI_4$ is highly uniform. This is a consequence of the PL being emitted mainly at the centre of the flake, where the energy of the exciton resonance is mainly determined by the effect of dielectric and quantum confinement within $(BA)_2PbI_4$.

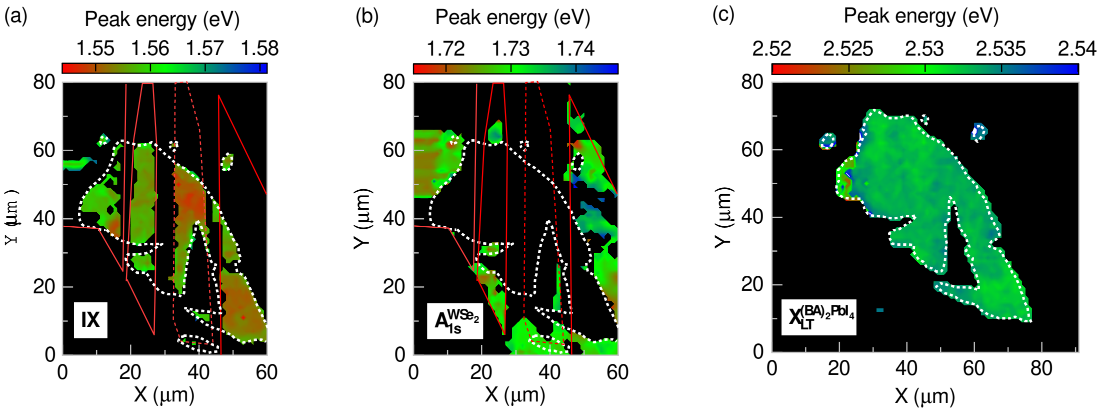

FIG. S2. Map of the PL energy of (a) the IX, (b) the intralayer WSe_2 exciton and (c) the intralayer exciton of the $(BA)_2PbI_4$ low temperature phase. The contour of monolayer WSe_2 is highlighted by continuous red lines, that of bilayer WSe_2 by dashed red lines. The $(BA)_2PbI_4$ flake is traced in a dashed white line.

III. DETAILED ASSIGNMENT OF PL AND REFLECTIVITY PEAKS

The differential reflectivity spectrum of WSe_2, shown in Fig. S3, consists of multiple resonances, assigned to 1s and 2s transitions of A ($A_{ns}^{WSe_2}$) and B ($B_{ns}^{WSe_2}$) excitons [10–13], as well as C exciton (C^{WSe_2}) [14–16]. The differential reflectivity of the $(BA)_2PbI_4$ flake presents a prominent resonance, which corresponds to the excitonic transition of the low temperature phase ($X_{LT}^{(BA)_2PbI_4}$) at ~ 2.54 eV. A weaker transition at ~ 2.38 eV, attributed to the exciton resonance of the high temperature phase ($X_{HT}^{(BA)_2PbI_4}$), can also be observed. The simultaneous observation of features related to both the low and high temperature phases has been reported for 2D perovskites deposited as thin films [17] or as exfoliated flakes [18]. It is explained by a partial inhibition of the phase transitions of small domains as a consequence of the strong contact between the perovskite flake and the substrate [18]. The recombination of the IX yields a single PL peak in the PL spectrum (see Fig. S3) with no additional feature attributable to the high-temperature phase [19]. This suggests that the fraction of the flake retaining the high temperature phase is small, and these microcrystalline inclusions do not affect the formation and recombination of the interlayer exciton.

The PL spectrum of the WSe_2 monolayer consists of the neutral exciton peak and a series of lower energy features attributed to the biexciton (XX^{WSe_2}) [20–23], triplet ($T_T^{WSe_2}$) and

singlet ($T_S^{WSe_2}$) charged excitons [23–25], and charged biexciton (XX^{-WSe_2}) [20–23], based on the energy separation with respect to the neutral exciton peak. The lowest energy peak originates from the recombination of localized excitons (L^{WSe_2}), which can contribute to the PL spectrum even after hBN encapsulation [20, 26]. The PL spectrum of $(BA)_2PbI_4$ is dominated by the recombination of the exciton in the low temperature phase, with a weak, low energy peak, which testifies the presence of inclusions of high temperature domains within the excitation spot. In the differential reflectivity spectrum of the heterostructure of Fig. S3, the resonances of $(BA)_2PbI_4$ are visible, while those of WSe_2 are strongly weakened and broadened. This occurs due to the presence of strong electronic interactions and non-radiative charge or energy transfer across the heterostructure [27, 28]. The most noticeable difference in the reflectivity spectrum of the heterostructure is a new feature, which we label X^{CT}, red-shifted with respect to the B exciton of WSe_2 of the isolated monolayer by $\sim 80\,\text{meV}$. This resonance is observed only on the heterostructure area.

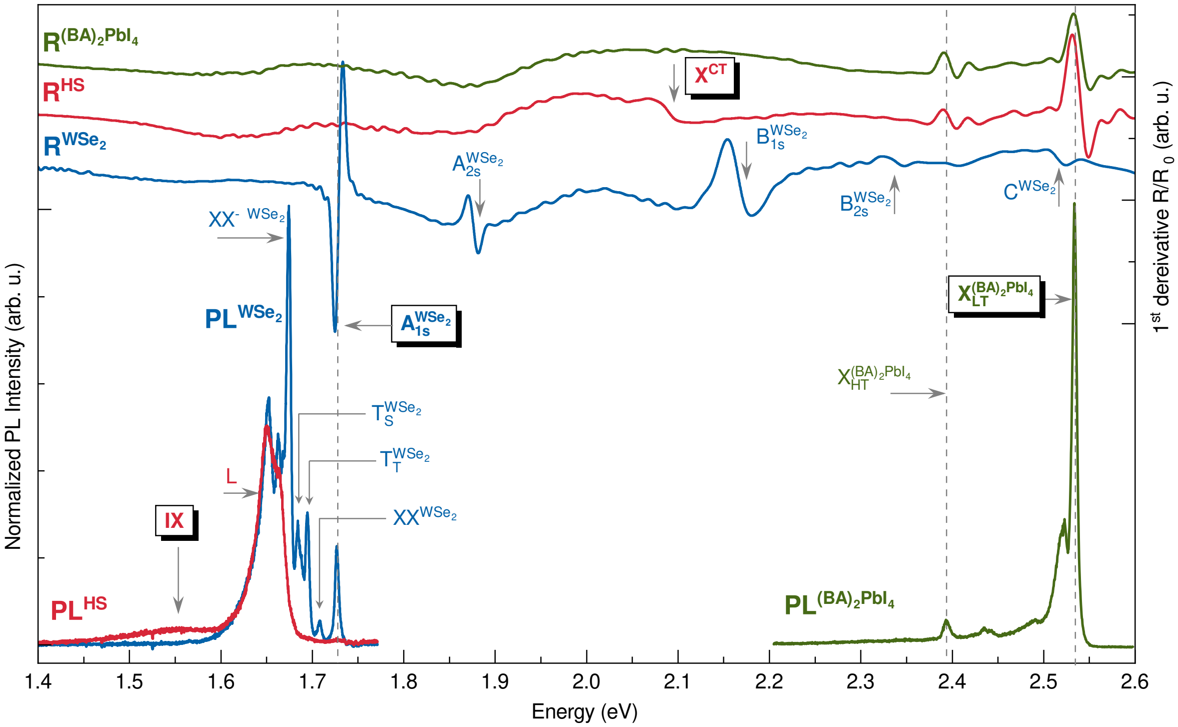

FIG. S3. Wide range μPL and first derivative of the reflectivity contrast spectra of isolated flakes and of the heterostructure (HS). The excitonic resonances are indicated by arrows.

IV. BAND STRUCTURE CALCULATIONS

Previous band structure calculations have demonstrated that 2D perovskite/TMD heterostructures are characterized by a type II band alignment [29–35]. However, to fully describe electronic properties, the heterostructure as a whole should be simulated [34, 35]. The calculated band structure of our heterostructure is shown in Fig. S4. This simulation reveals that the edge of the conduction band is dominated by states belonging to the TMD monolayer. In the conduction band, the states of the organic barriers are energetically higher than those of both the TMD and the PbI_4 octahedral units, as schematically illustrated in Fig. 1(a,c). Thus, the organic spacer acts as a barrier for electron transfer in the conduction band. In contrast, the edge of the valence band is mainly contributed by the states of PbI_4. Moreover, a closer inspection to the projected density of states of Fig. S4 reveals the presence of a non-vanishing contribution of states related to organic spacers at energies intermediate between those of PbI_4 and of the TMD monolayer close to the valence band edge. This leads to the cascaded band alignment in the valence band, also depicted in the inset of Fig. 1(a,c) of the main text, which favours the hole transfer from WSe_2 to PbI_4 [34, 35]. The spatial separation of a photocreated electron-hole pair that results from this band alignment represents the prerequisite for the formation of the IX.

V. EXCITATION POWER DEPENDENCE OF THE PL SPECTRUM

An additional argument to support the interlayer origin of the low energy PL peak comes from its excitation power dependence. PL spectra measured at different excitation powers are shown in Fig. S5(a). The IX peak displays a notable blue shift with increasing power, which can be better appreciated in the close-up spectra shown in the inset of Fig. S5(a). By fitting the PL spectrum with Gaussian peaks, we extracted the intensity of the intralayer WSe_2 exciton and of the IX as a function of the excitation power, which we show in Fig. S5(b). These trends can be modelled by a power law ($I \propto P^b$), which yields an exponent $b \sim 1$ for both intralayer exciton and IX [36]. This suggests that both these lines originate from the recombination of a single Coulomb bound electron-hole pair. The power used to excite the PL of the heterostructure was limited to 50 µW, which did not allow us to reach the saturation level sometimes reported for IXs [37, 38]. The power dependence of the IX energy

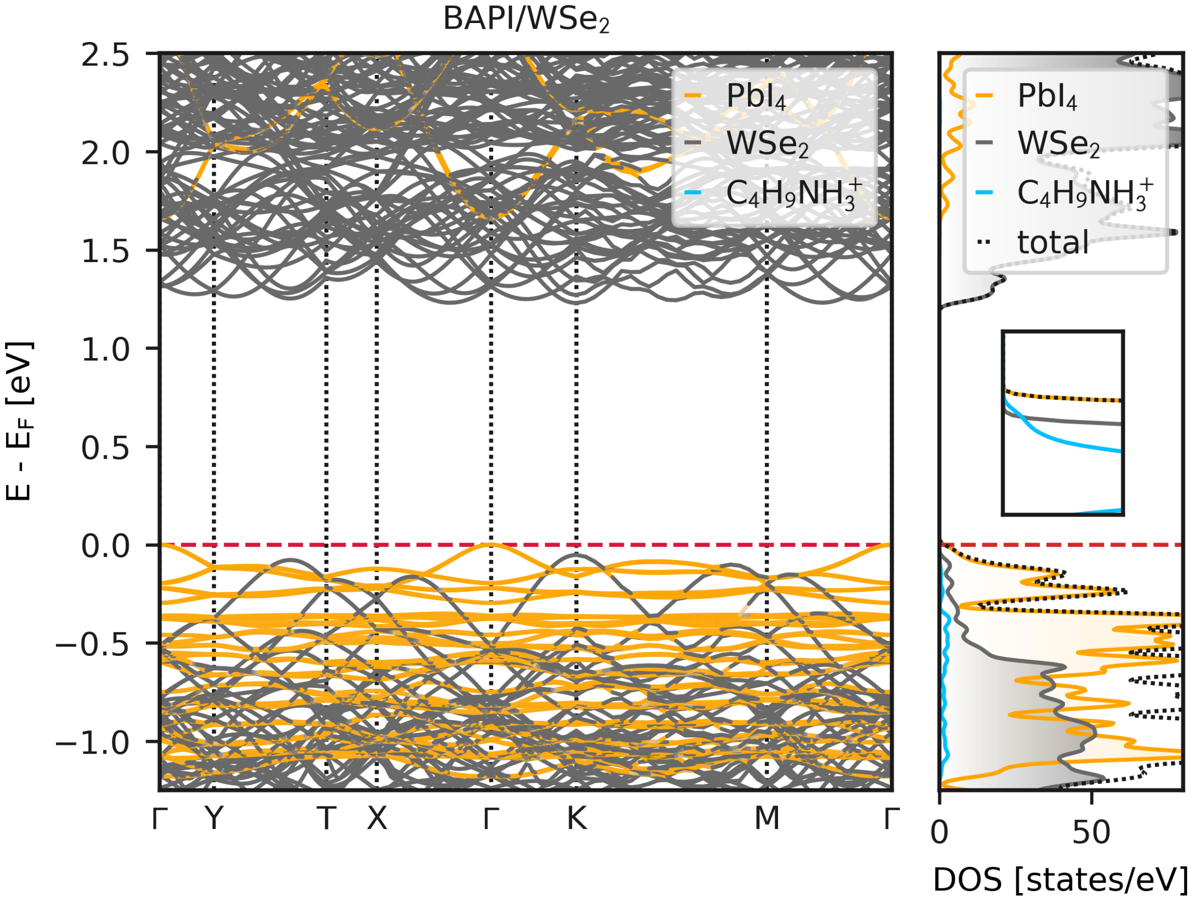

FIG. S4. (Left) Mulliken-projected band structure and (right) projected density of state (PDOS) of the $(BA)_2PbI_4/WSe_2$ monolayer heterostructure. The majority contributions to the electronic states are shown by colour-coding the specific building block mainly responsible for that state. The Fermi level is shifted to the top of valence band and set at zero (horizontal red dashed line). The top of the valence band is dominated by PbI_4 states, while the bottom of the conduction band by WSe_2 states. The inset in the PDOS shows a close-up view of the region close to the Fermi level, highlighting the states from the organic spacer between those of PbI_4 and WSe_2.

is summarized in Fig. S5(c). The approximately linear blue shift with increasing excitation power we observed reflects the expected behaviour of spatially indirect excitons in coupled epitaxial quantum wells [39], TMD heterobilayers [30, 40–42], and 2D perovskite/TMD heterostructures [8, 19, 43, 44]. This blue shift is connected to the out-of-plane dipole moment of IXs, wherein the electron and the hole are spatially separated in two different materials due to the type II band alignment, in combination with an increased density at

high excitation power [45]. Thus, the blue shift of the low energy peak with increasing excitation power, characteristic of the dipole-dipole repulsion, confirms its interlayer nature.

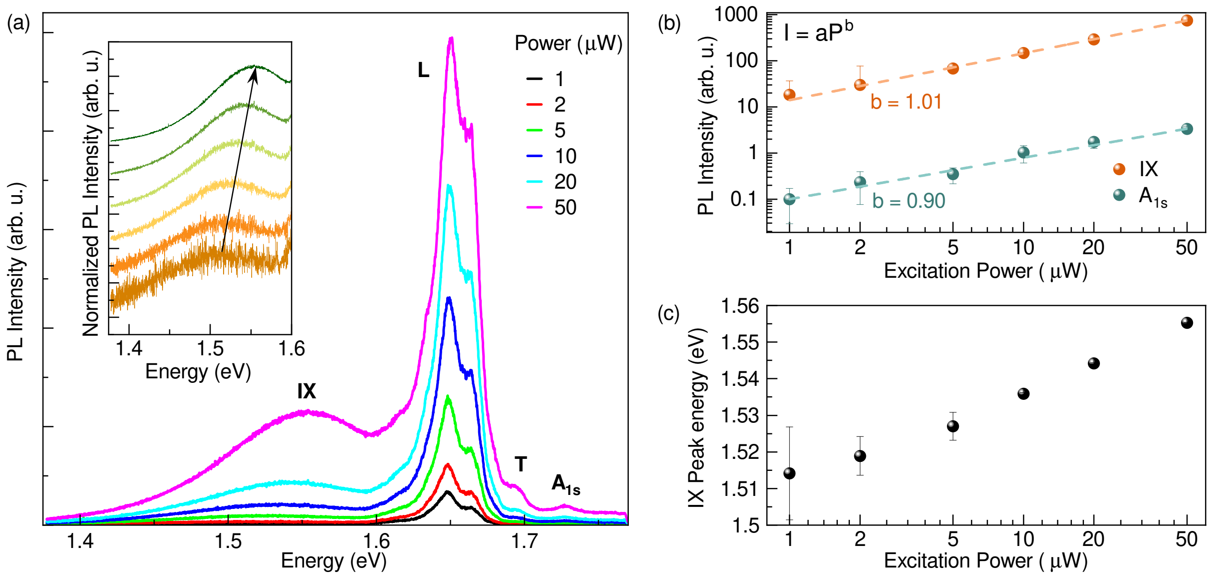

FIG. S5. (a) Main: Power dependent μPL spectrum of the $(BA)_2PbI_4/WSe_2$ heterostructure. Inset: close-up on the interlayer exciton (IX) transition. Spectra in the inset are normalized and shifted vertically for better visibility. The arrow showcases the blue shift exhibited by this peak with increasing excitation power. (b) PL intensity of the WSe_2 A_{1s} exciton and of the IXs as a function of the excitation power. The dashed lines are fits to a power law. The exponent b of the power law extracted from the fit are indicated. (c) Energy of the IX as a function of the excitation power.

VI. TEMPERATURE DEPENDENCE OF THE PL SPECTRUM

The temperature dependence of the PL spectrum can provide complementary information about the origin of the peaks. We compare the salient features of the temperature dependence of the intralayer neutral exciton of WSe_2 and of the IX. The PL spectra of the heterostructure measured at temperatures ranging from 10 K to 270 K are shown in Fig. S6(a). The PL is excited below the bandgap of $(BA)_2PbI_4$ to minimize the potentially disrupting influence from the low energy tail of the PL spectrum of the 2D perovskite flake. The temperature dependence of intralayer exciton species displays all the expected features

for the PL spectrum of WSe_2 monolayers. All peaks red shift with increasing temperature. The temperature dependence of the energy of the intralayer neutral exciton is summarized in Fig. S6(b). This data is fitted with a function customarily used to analyze the temperature dependent band gap $E(T)$ of layered semiconductors [46–49]:

$$E(T) = E(0) - S\langle\hbar\omega\rangle \left(\coth \frac{\langle\hbar\omega\rangle}{2\mathrm{k_B}T} - 1\right), \tag{S1}$$

where $E(0)$ is the zero-temperature energy of the transition, S is a parameter which describes phenomenologically the strength of the electron-phonon coupling and $\langle\hbar\omega\rangle$ represents an effective acoustic phonon frequency involved in the electron-phonon interaction. Our fit of the energy of the intralayer exciton yields $S \sim 2.15$ and $\langle\hbar\omega\rangle \sim 17.5\,\mathrm{meV}$, which are values comparable to those reported previously [49]. Interestingly, the IX peak displays a more pronounced decrease of the energy with increasing temperature temperature, which results in $S \sim 7.7$ and $\langle\hbar\omega\rangle \sim 29.4\,\mathrm{meV}$. The more pronounced red shift of the IX is due to the combined contribution of the temperature dependence of the WSe_2 conduction band and of the $(BA)_2PbI_4$ valence band. The larger S and $\langle\hbar\omega\rangle$ found in the case of the IX are indicative of the contribution of the $(BA)_2PbI_4$ valence band to the interlayer transition. In 2D perovskites, the electron-phonon coupling is considerably stronger than in other semiconductors [50], due to the pronounced ionicity of the lead halide perovskite lattice [51].

An additional confirmation of the strong influence of both constituents on the properties of the IX can be found in the temperature dependence of its PL intensity. Looking at the PL shown in Fig. S6(a), we notice that the low energy peaks associated with bound excitons are rapidly quenched with increasing temperature [52]. The intensity of the charged exciton peaks initially displays an increase with increasing temperature, and then is quenched at higher temperatures [52]. We focus here on the temperature dependence of the intralayer neutral exciton and of the IX. In Fig. S6(a), one can notice that the intensity of the intralayer neutral exciton strongly increases with increasing temperature even when all other lower energy transitions have disappeared. This is observed in systems in which the lowest-lying exciton state is optically dark. Part of the population of this optically inactive state is thermally excited to the higher energy optically bright state, which leads to a stronger PL at higher temperatures [53]. Importantly, a very similar trend is globally exhibited by the IX. This mirroring behaviour highlights how carriers that relax to the IX state originate

from the conduction band of WSe_2 and therefore follow the trends set by this material.

We fitted the temperature dependence of the intensity of the intralayer excitons and of the IX with a modified Arrhenius formula, used when an emissive state is supplied by a finite carrier reservoir [49, 54]

$$I(T) = I_0 \frac{1 + B_\mathrm{s} \mathrm{e}^{-\frac{E_\mathrm{s}}{\mathrm{k_B}T}}}{1 + B_\mathrm{q} \mathrm{e}^{-\frac{E_\mathrm{q}}{\mathrm{k_B}T}}}, \tag{S2}$$

where B_s (B_q) is related to the ratio of radiative to non-radiative lifetimes of carriers which supply (are lost from) the emissive state [55], E_s represents the activation energy of the processes which lead to the increase of the PL intensity, and E_q designates the activation energy of the PL thermal quenching. Using Eq. (S2), we extracted the activation energies of the reservoir of the intralayer excitons and IXs, which amount to 27 meV and 15 meV, respectively. These energies do not necessarily correspond to the energy separation between dark and bright intralayer excitons, because this analysis does not account for competing non-radiative recombination channels, for the temperature dependence of the radiative recombination rate and for the presence of a thermal equilibrium between the different populations [53]. The lower activation energy of the thermal quenching of the IX compared to that of the intralayer exciton (61 meV versus 91 meV) could be related to the lower binding energy of the IX and the presence of additional non-radiative recombination paths related to the quality of the TMD-2D perovskite interface, which instead likely influences less the PL of intralayer excitons.

VII. TIME-RESOLVED PHOTOLUMINESCENCE OF INTERLAYER EXCITON

We show in Fig. S7 the time-resolved PL signal of the IX measured at 80 K. The decay is characterized by a fast and a slow component, which we evaluated with a biexponential fit to be $\tau_1 = 1.74$ ns and $\tau_2 = 9.70$ ns, respectively. These decay times exceed those typical of both TMD monolayers, which are in the range of a few picoseconds [52], and those of $(BA)_2PbI_4$, which are in the range of a nanosecond [56]. This long life time is consistent with the large electron-hole spatial separation typical of IX species [37], and has already been reported in similar heterostructures [8, 19, 43, 44].

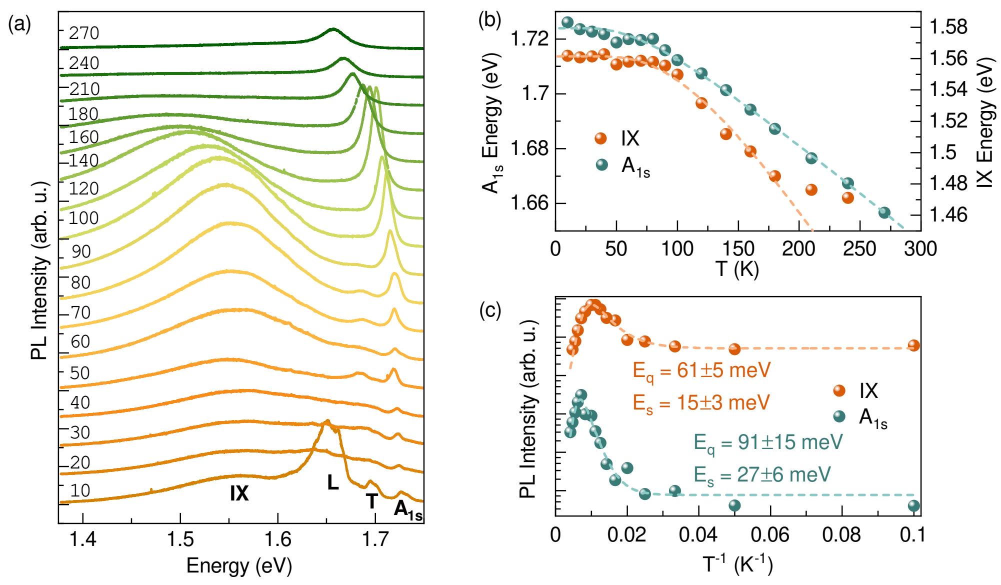

FIG. S6. (a) PL spectrum of the heterostructure excited below the $(BA)_2PbI_4$ bandgap measured at the temperatures indicated on the left of the panel. A_{1s} identifies the PL peak related to the recombination of the free intralayer exciton, T labels the charged excitons, L the localized excitons and IX the interlayer exciton. (b) Energy of the intralayer exciton (blue points) and of the IX (red points) as a function of the temperature. The lines are fits to the model of Eq. (S1). (c) Arrhenius plot of the intralayer exciton (blue points) and of the IX (red points) as a function of the inverse temperature. The lines are fits to the modified Arrhenius model detailed in Eq. (S2).

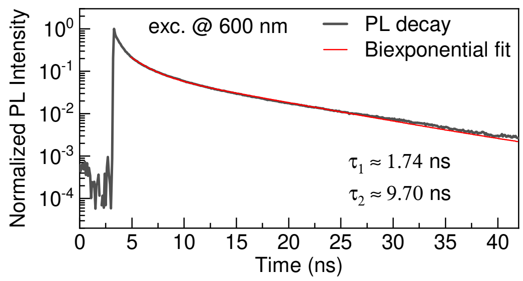

FIG. S7. Time-resolved photoluminescence of the IX measured at 80 K. The red curve shows the biexponential fitting of the decay. The two extracted decay times are indicated.

VIII. POSSIBLE ALTERNATIVE ORIGINS OF IX AND X^{CT} PEAKS

In the literature, the presence of additional peaks in the optical spectra of lead halide perovskites, TMD monolayers, and their heterostructures has been attributed to multiple

mechanisms. These include energy transfer, defect-related states, strain fields, and self-trapped excitons. We show here that these can be ruled out as responsible for the observation of the IX and X^{CT} peaks.

Energy transfer

Energy transfer is generally considered to lead to enhancement of the PL intensity of one of the components when the other component of the heterostructure is excited, as shown for the PL of the WS_2 monolayer part of a $WS_2/(PEA)_2PbI_4$ heterostructure [29]. The PL maps in Fig. S1(c), measured using an excitation laser at an energy greater than the optical band gap of $(BA)_2PbI_4$, instead demonstrate that the intensity of the WSe_2 PL is suppressed in the heterostructure area. This points to a negligible contribution of the energy transfer in this heterostructure. During our circular polarization resolved PLE measurements, the heterostructure was excited by keeping the excitation laser energy below the exciton resonance of $(BA)_2PbI_4$, as explained in the Methods section. As a consequence, there can be no Förster-like energy transfer from the 2D perovskite to the TMD monolayer. We conclude that in the experimental conditions used in the polarization resolved PLE measurements of Fig. 2, the energy transfer is not relevant. Finally, we note that for a Föster type energy transfer to occur efficiently, there should be a good energy match between the relevant exciton levels. In this heterostructure, the energy of the $(BA)_2PbI_4$ exciton is significantly larger than that of both A and B excitons of WSe_2 [35], which additionally proves that energy transfer is expected to play a minor role as the origin of the interlayer exciton peaks observed in the optical spectra.

To confirm this, the most commonly used experimental technique is PL excitation (PLE) spectroscopy [57]. In the presence of energy transfer, tuning the excitation laser in resonance with the excitonic transition of one of the materials part of the heterostructure leads to enhanced PL of the other material [29, 34, 35]. We performed additional PLE measurements without resolving the signal in the circular polarization basis, always exciting below the optical band gap of $(BA)_2PbI_4$. The intensity of the IX peak increases when the laser is scanned through both the WSe_2 B exciton resonance and the X^{CT} (see S8(a)). In contrast, the intensity of the 1s exciton of the WSe_2 monolayer only increases when the laser is resonant with the B exciton resonance. This is consistent with the observation of Fig. 2(b). This result

confirms the negligible role of the energy transfer in determining the optoelectronic properties of this heterostructure. The lack of enhancement of the WSe_2 PL when the excitation laser is resonant with X^{CT} can be understood by considering the energy landscape probed by the charge carriers, depicted schematically in Fig. S8(c). Resonant excitation of X^{CT} induces the predominant formation of electron-hole pairs at this transition. The schematic highlights how the hole relaxes towards the most energetically favourable state, represented by the valence band contributed by the PbI_4 slab.

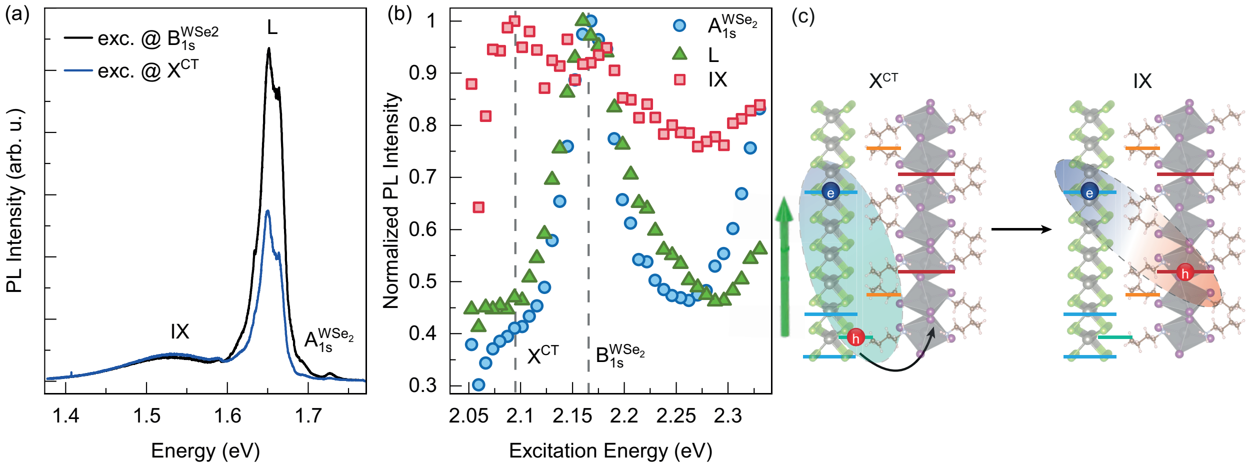

FIG. S8. (a) PL spectrum of the heterostructure excited in resonance with the B exciton of WSe_2 and with the interlayer charge transfer exciton X^{CT}. (b) Intensity of the PL of the intralayer and localized exciton of WSe_2 ($A_{1s}^{WSe_2}$ and L, respectively) and of the interlayer exciton IX. (c) Schematic of the relaxation process in the single particle picture after resonant excitation of X^{CT}.

Defect-related and self-trapped exciton emission

While the presence of defect-related states plays an important role in these materials in general [58–61], we can exclude that they are mainly involved in the formation and properties of the interlayer exciton species IX and X^{CT} of our heterostructure. First, both peaks are seen only in the heterostructure area. This strongly suggests that they are not inherent features of the individual materials and therefore cannot be associated with defects or self-trapping phenomena. Concerning X^{CT}, this peak can be clearly resolved in reflectivity contrast measurements. Because the weak absorption related to defect-bound exciton generally does not yield any feature in the reflectivity spectrum [14, 59, 61–63], we can conclude that

X^{CT} should not be attributed to defect-related transitions. The observation of a resonance associated to X^{CT} in the reflectivity contrast spectrum enables us to exclude self-trapped excitons as the origin of this feature. Self-trapped excitons are transient species that cannot be excited directly but result from the interaction of a free exciton with a polar lattice [61, 64]. Therefore, they are not expected to yield any feature in the reflectivity contrast spectrum. Moreover, very recent results clearly demonstrate that self-trapped excitons are not responsible for the low energy peaks in the optical spectra of $(BA)_2PbI_4$ [60], which is an additional reason to rule out their influence.

Concerning the IX, multiple features prevent its association with defect-related transitions and self-trapped excitons. The strong blue shift of its PL peak with increasing excitation power unambiguously points to its dipolar character, as discussed above. This excludes the contribution of defect-assisted transitions or self trapping to the IX peak. Additionally, the linear power dependence of its PL intensity and the lack of saturation are in contradiction to the typical behaviour exhibited by peaks related to defect-bound excitons [29, 65]. The non-monotonic temperature dependence of the IX PL intensity is in contrast with the decrease of the PL intensity that self-trapped excitons generally exhibit with increasing temperature [66–69]. Finally, as an additional proof that IX and X^{CT} are not related to defect states or self-trapped excitons, we show the PL spectrum of the isolated $(BA)_2PbI_4$ flake and of the $(BA)_2PbI_4/WSe_2$ heterostructure over a wider spectral range in Fig. S9(a,b), respectively. In both PL spectra, we note a low energy peak at energies different from those of IX and X^{CT}, likely associated with defects of $(BA)_2PbI_4$ [60]. This further demonstrates how the IX and X^{CT} transitions are inherently and solely related to interlayer exciton species of the heterostructure.

Strain

As discussed in the Methods section, the heterostructure is fabricated by mechanical exfoliation of the individual flakes, followed by all-dry transfer of the flakes to the target substrate by means of a viscoelastic stamp [1]. Given that both components of the heterostructure are van der Waals materials, the interaction at their interface is not as strong as that between the epilayer and the substrate during epitaxial growth. This strongly reduces any impact of the lattice mismatch on the electronic properties of the heterostructure

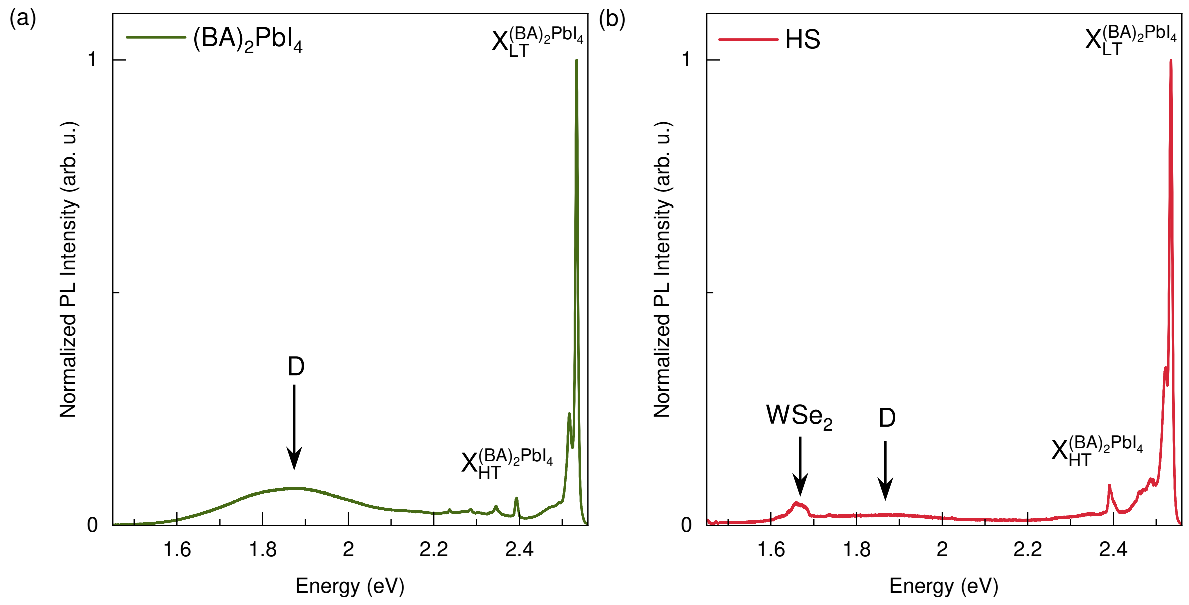

FIG. S9. PL spectrum of (a) isolated $(BA)_2PbI_4$ flake and (b) $(BA)_2PbI_4/WSe_2$ monolayer heterostructure shown over a wide spectral range.

[70]. Thus, the flakes in the heterostructure cannot be subjected to a strain that would be large enough to induce shifts that would correspond to the energies of IX and X^{CT} with respect to the excitonic resonances of WSe_2 and $(BA)_2PbI_4$, respectively. As a consequence, we expect that the fabrication of the heterostructure leads to a negligible amount of strain, in spite of the significant differences in the crystal structures and the lattice parameters. Moreover, the values of strain or hydrostatic pressure that would be needed to shift the exciton of $(BA)_2PbI_4$ by $\approx 440\,\mathrm{meV}$ (energy difference between X^{CT} and the exciton of $(BA)_2PbI_4$) would be more than 10 GPa (estimated based on the hydrostatic pressure gauge of 35 meV/GPa [71]) or more than 10% strain (which would likely lead to the fracture of the flake), if we take into account the strain gauge reported for 2D perovskites [72]. Similarly, to induce a shift of 155 meV, corresponding to the energy difference between the IX and the exciton of WSe_2, the WSe_2 flake should be subjected to a strain greater than 2.8%, based on the typical strain gauge reported in the literature [73]. It is even less likely that the (virtually negligible) strain of one of the components of the heterostructure leads to the formation of new states. These are typically observed at hydrostatic pressures larger than 1.5 GPa [74].

IX. PUMP-PROBE REFLECTIVITY MAPS

Fig. S10(a) illustrates the transient reflectivity $\Delta R/R$ maps as a function of the pump-probe temporal delay and the probe energy measured on the WSe_2 monolayer (ΔR is the transient variation of reflectivity upon the pump excitation, while R indicates the static reflectivity). For both the measurements the energy of the pump is tuned above the quasi-particle bandgap of both the constituents of the heterostructure. The $\Delta R/R$ map of the WSe_2 displays a strong pump-probe signal around the energy of the A exciton. At higher probe energies one can also identify the transient signals at the energies of the 2s and B excitons. In Fig. S10(b), we show the transient reflectivity map of the heterostructure. Although the WSe_2 resonances are still present, they are broadened and have lower intensity compared to those on the isolated WSe_2, due to the presence of additional non-radiative recombination channels, such as charge transfer [75]. An additional transient signal appears at the energy of 2.08 eV, assigned to the X^{CT} resonance.

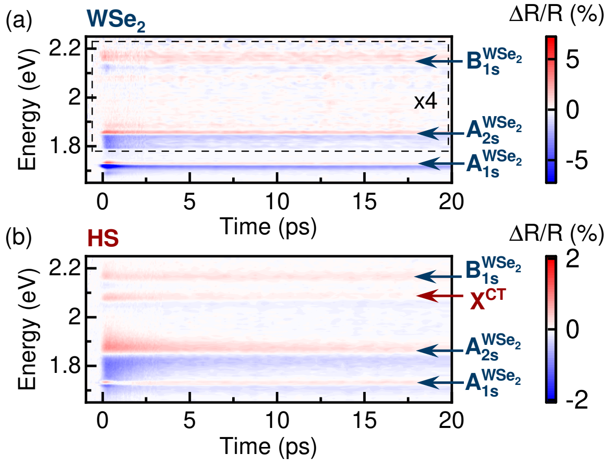

FIG. S10. Transient differential reflectivity map excited at 2.64 eV (above the perovskite band gap) as a function of the energy and of the pump-probe delay of (a) isolated WSe_2 monolayer and of (b) $(BA)_2PbI_4/WSe_2$ heterostructure. In (a), the high energy part of the transient spectrum has been rescaled to highlight the absence of other resonances than WSe_2 intralayer excitons. The excitonic resonances are indicated. The resonance corresponding to the WSe_2 A exciton ($A_{1s}^{WSe_2}$) has been rescaled for increased clarity.

X. DYNAMICS OF CHARGE TRANSFER

To gain more insight into the mechanisms that lead to the ultrafast formation of the IX, we measure the interlayer charge transfer dynamics using broadband pump-probe optical microscopy measurements. In this experiment, the sample is photoexcited by an ultra-short laser pulse (duration of $\sim$ 100 fs), while the transient reflectivity spectrum $\Delta R/R$ is measured as a function of the temporal delay and the probe spectral range. In Fig. S11, we summarize the results obtained when the pump laser pulse is tuned in resonance with the 1s absorption peak of the WSe_2 monolayer $A_{1s}^{WSe_2}$, which is below the band gap of $(BA)_2PbI_4$. In Fig. S11(a), we show the differential reflectivity spectrum measured on the isolated $(BA)_2PbI_4$ flake as a function of the energy and of the pump-probe delay. The lack of a transient response is consistent with the fact that excitation is performed in the transparency window. The faint signal detected at energies corresponding to the exciton resonance of the low-temperature phase of $(BA)_2PbI_4$ is likely related to the two-photon absorption of the 2D perovskite flake. The situation is radically different when the differential reflectivity spectrum is acquired in the heterostructure area, as shown in Fig. S11(b). Although the excitation is performed well below the band gap, we notice a distinct resonance at the energy of the $(BA)_2PbI_4$ exciton. The substantial difference between the optical responses of the heterostructure and of the isolated $(BA)_4PbI_4$ flake can be nicely seen by comparing their transient reflectivity spectra extracted at a delay of 5 ps, which are shown in Fig. S11(c). While the differential reflectivity of the isolated 2D perovskite flake is featureless, the transient spectrum measured on the heterostructure shows a pronounced signal at the energy of the exciton of the low temperature phase $X_{LT}^{(BA)_2PbI_4}$ and an additional broad signal at energies close the resonance associated to the high temperature phase of $(BA)_4PbI_4$ $X_{HT}^{(BA)_2PbI_4}$ frozen at low temperature [17, 18]. The observation of these additional features is a direct proof of the interlayer hole transfer from the WSe_2 monolayer. The build-up of the $X_{LT}^{(BA)_2PbI_4}$ bleaching signal in Fig. S11 is shorter than the instrument response function, which allows us to set an upper limit of $\sim$ 100 fs to the charge transfer time.

---

[1] A. Castellanos-Gomez, M. Buscema, R. Molenaar, V. Singh, L. Janssen, H. S. Van Der Zant, and G. A. Steele, Deterministic transfer of two-dimensional materials by all-dry viscoelastic

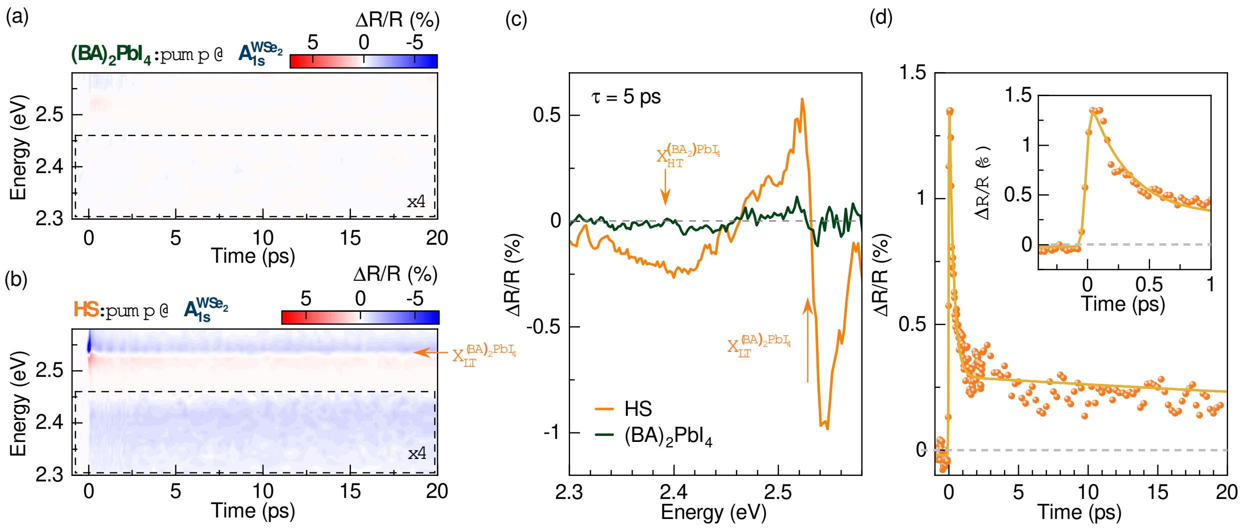

FIG. S11. Transient differential reflectivity map excited in resonance with the A exciton of WSe_2 $A_{1s}^{WSe_2}$ as a function of the energy and of the pump-probe delay of (a) bare $(BA)_2PbI_4$ flake and of (b) $(BA)_2PbI_4/WSe_2$ heterostructure. The low energy part of the transient spectrum has been rescaled to highlight the absence of transitions. (c) Differential reflectivity spectrum of the heterostructure and of the bare $(BA)_2PbI_4$ flake extracted at a delay $\tau = 5\,\text{ps}$. The excitonic resonances are indicated. (d) Main: transient differential reflectivity of the excitonic transition of the low temperature phase measured on the heterostructure ($X_{LT}^{(BA)_2PbI_4}$) as a function of the pump-probe delay. Inset: Blow-up of the rise of the pump-probe signal of the $(BA)_2PbI_4$ exciton to highlight its instantaneous build-up following charge transfer. The line is the fit of an exponential rise and a bi-exponential decay model to the experimental data.

stamping, 2D Materials **1**, 011002 (2014).

[2] R. Kempt, romankempt/hetbuilder: Zenodo Release (2021).

[3] V. Blum, R. Gehrke, F. Hanke, P. Havu, V. Havu, X. Ren, K. Reuter, and M. Scheffler, Ab initio molecular simulations with numeric atom-centered orbitals, Computer Physics Communications **180**, 2175 (2009).

[4] J. P. Perdew, K. Burke, and M. Ernzerhof, Generalized gradient approximation made simple, Physical Review Letters **77**, 3865 (1996).

[5] A. Tkatchenko, A. Ambrosetti, and R. A. DiStasio, Interatomic methods for the dispersion energy derived from the adiabatic connection fluctuation-dissipation theorem, The Journal of Chemical Physics **138**, 074106 (2013).

[6] J. Hermann and A. Tkatchenko, Density functional model for van der waals interactions: Unifying many-body atomic approaches with nonlocal functionals, Physical Review Letters **124**, 146401 (2020).

[7] A. Genco, C. Trovatello, C. Louca, K. Watanabe, T. Taniguchi, A. I. Tartakovskii, G. Cerullo, and S. Dal Conte, Ultrafast exciton and trion dynamics in high-quality encapsulated MoS_2 monolayers, physica status solidi (b) **260**, 2200376 (2023).

[8] D. Yang, J. Hu, Y. Chen, and D. Li, Organic cation modulation of interlayer exciton emission in two-dimensional perovskite/monolayer transition metal dichalcogenide heterostructures, Advanced Optical Materials **11**, 2300398 (2023).

[9] A. Raja, A. Chaves, J. Yu, G. Arefe, H. M. Hill, A. F. Rigosi, T. C. Berkelbach, P. Nagler, C. Schüller, T. Korn, N. Colin, J. Hone, L. E. Brus, T. F. Heinz, D. R. Reichmann, and A. Chernikov, Coulomb engineering of the bandgap and excitons in two-dimensional materials, Nature Communications **8**, 15251 (2017).

[10] M. Manca, M. M. Glazov, C. Robert, F. Cadiz, T. Taniguchi, K. Watanabe, E. Courtade, T. Amand, P. Renucci, X. Marie, G. Wang, and B. Urbaszek, Enabling valley selective exciton scattering in monolayer WSe_2 through upconversion, Nature Communications **8**, 14927 (2017).

[11] A. V. Stier, N. P. Wilson, K. A. Velizhanin, J. Kono, X. Xu, and S. A. Crooker, Magnetooptics of exciton Rydberg states in a monolayer semiconductor, Physical Review Letters **120**, 057405 (2018).

[12] S.-Y. Chen, T. Goldstein, J. Tong, T. Taniguchi, K. Watanabe, and J. Yan, Superior valley polarization and coherence of 2s excitons in monolayer WSe_2, Physical Review Letters **120**, 046402 (2018).

[13] M. Molas, A. Slobodeniuk, K. Nogajewski, M. Bartos, Ł. Bala, A. Babiński, K. Watanabe, T. Taniguchi, C. Faugeras, and M. Potemski, Energy spectrum of two-dimensional excitons in a nonuniform dielectric medium, Physical Review Letters **123**, 136801 (2019).

[14] A. Arora, M. Koperski, K. Nogajewski, J. Marcus, C. Faugeras, and M. Potemski, Excitonic resonances in thin films of WSe_2: from monolayer to bulk material, Nanoscale **7**, 10421 (2015).

[15] A. Hanbicki, M. Currie, G. Kioseoglou, A. Friedman, and B. Jonker, Measurement of high exciton binding energy in the monolayer transition-metal dichalcogenides WS_2 and WSe_2, Solid State Communications **203**, 16 (2015).

[16] R. Frisenda, Y. Niu, P. Gant, A. J. Molina-Mendoza, R. Schmidt, R. Bratschitsch, J. Liu, L. Fu, D. Dumcenco, A. Kis, D. Perez de Lara, and A. Castellanos-Gomez, Micro-reflectance and transmittance spectroscopy: a versatile and powerful tool to characterize 2D materials, Journal of Physics D: Applied Physics **50**, 074002 (2017).

[17] M. Baranowski, S. J. Zelewski, M. Kepenekian, B. Traoré, J. M. Urban, A. Surrente, K. Galkowski, D. K. Maude, A. Kuc, E. P. Booker, R. Kudrawiec, S. D. Stranks, and P. Plochocka, Phase-transition-induced carrier mass enhancement in 2D Ruddlesden–Popper perovskites, ACS Energy Letters **4**, 2386 (2019).

[18] O. Yaffe, A. Chernikov, Z. M. Norman, Y. Zhong, A. Velauthapillai, A. Van Der Zande, J. S. Owen, and T. F. Heinz, Excitons in ultrathin organic-inorganic perovskite crystals, Physical Review B **92**, 045414 (2015).

[19] Z. Liu, Y. Chen, J. Li, W. Yao, T. Luo, and D. Li, Probing local structural phase transition at the surface of $(BA)_2PbI_4$ via interlayer exciton emission, Advanced Functional Materials **34**, 2312074 (2024).

[20] Z. Ye, L. Waldecker, E. Y. Ma, D. Rhodes, A. Antony, B. Kim, X.-X. Zhang, M. Deng, Y. Jiang, Z. Lu, D. Smirnov, K. Watanabe, T. Taniguchi, J. Hone, and T. F. Heinz, Efficient generation of neutral and charged biexcitons in encapsulated WSe_2 monolayers, Nature Communications **9**, 3718 (2018).

[21] S.-Y. Chen, T. Goldstein, T. Taniguchi, K. Watanabe, and J. Yan, Coulomb-bound four-and five-particle intervalley states in an atomically-thin semiconductor, Nature Communications **9**, 3717 (2018).

[22] Z. Li, T. Wang, Z. Lu, C. Jin, Y. Chen, Y. Meng, Z. Lian, T. Taniguchi, K. Watanabe, S. Zhang, D. Smirnov, and S.-F. Shi, Revealing the biexciton and trion-exciton complexes in BN encapsulated WSe_2, Nature Communications **9**, 3719 (2018).

[23] M. Barbone, A. R.-P. Montblanch, D. M. Kara, C. Palacios-Berraquero, A. R. Cadore, D. De Fazio, B. Pingault, E. Mostaani, H. Li, B. Chen, K. Watanabe, T. Taniguchi, S. Tongay, G. Wang, A. C. Ferrari, and M. Atatüre, Charge-tuneable biexciton complexes in monolayer WSe_2, Nature Communications **9**, 3721 (2018).

[24] E. Courtade, M. Semina, M. Manca, M. Glazov, C. Robert, F. Cadiz, G. Wang, T. Taniguchi, K. Watanabe, M. Pierre, W. Escoffier, E. Ivchenko, P. Renucci, X. Marie, T. Amand, and B. Urbaszek, Charged excitons in monolayer WSe_2: Experiment and theory, Physical Review

B **96**, 085302 (2017).

[25] Z. Li, T. Wang, Z. Lu, M. Khatoniar, Z. Lian, Y. Meng, M. Blei, T. Taniguchi, K. Watanabe, S. A. McGill, S. Tongay, V. M. Menon, D. Smirnov, and S.-F. Shi, Direct observation of gate-tunable dark trions in monolayer WSe_2, Nano Letters **19**, 6886 (2019).

[26] F. Cadiz, E. Courtade, C. Robert, G. Wang, Y. Shen, H. Cai, T. Taniguchi, K. Watanabe, H. Carrere, D. Lagarde, M. Manca, T. Amand, P. Renucci, S. Tongay, X. Marie, and B. Urbaszek, Excitonic linewidth approaching the homogeneous limit in MoS_2-based van der Waals heterostructures, Physical Review X **7**, 021026 (2017).

[27] L. Wu, Y. Chen, H. Zhou, and H. Zhu, Ultrafast energy transfer of both bright and dark excitons in 2D van der Waals heterostructures beyond dipolar coupling, ACS Nano **13**, 2341 (2019).

[28] H. Zhou, Y. Zhao, W. Tao, Y. Li, Q. Zhou, and H. Zhu, Controlling exciton and valley dynamics in two-dimensional heterostructures with atomically precise interlayer proximity, ACS Nano **14**, 4618 (2020).

[29] Q. Zhang, E. Linardy, X. Wang, and G. Eda, Excitonic energy transfer in heterostructures of quasi-2D perovskite and monolayer WS_2, ACS Nano **14**, 11482 (2020).

[30] Y. Chen, Z. Liu, J. Li, X. Cheng, J. Ma, H. Wang, and D. Li, Robust interlayer coupling in two-dimensional perovskite/monolayer transition metal dichalcogenide heterostructures, ACS Nano **14**, 10258 (2020).

[31] Y. Chen, J. Ma, Z. Liu, J. Li, X. Duan, and D. Li, Manipulation of valley pseudospin by selective spin injection in chiral two-dimensional perovskite/monolayer transition metal dichalcogenide heterostructures, ACS Nano **14**, 15154 (2020).

[32] Q. Wang, Q. Zhang, X. Luo, J. Wang, R. Zhu, Q. Liang, L. Zhang, J. Z. Yong, C. P. Yu Wong, G. Eda, J. H. Smet, and A. T. Wee, Optoelectronic properties of a van der Waals WS_2 monolayer/2D perovskite vertical heterostructure, ACS Applied Materials & Interfaces **12**, 45235 (2020).

[33] H. Zhou, H. Lai, X. Sun, N. Zhang, Y. Wang, P. Liu, Y. Zhou, and W. Xie, Van der Waals MoS_2/two-dimensional perovskite heterostructure for sensitive and ultrafast sub-band-gap photodetection, ACS Applied Materials & Interfaces **14**, 3356 (2022).

[34] M. Karpinska, M. Liang, R. Kempt, K. Finzel, M. Kamminga, M. Dyksik, N. Zhang, C. Knodlseder, D. K. Maude, M. Baranowski, Ł. Kłoptowski, J. Ye, A. Kuc, and P. Plochocka,

Nonradiative energy transfer and selective charge transfer in a WS_2/$(PEA)_2PbI_4$ heterostructure, ACS Applied Materials & Interfaces **13**, 33677 (2021).

[35] M. Karpińska, J. Jasiński, R. Kempt, J. Ziegler, H. Sansom, T. Taniguchi, K. Watanabe, H. Snaith, A. Surrente, M. Dyksik, D. Maude, Ł. Kłoptowski, A. Chernikov, A. Kuc, M. Baranowski, and P. Plochocka, Interlayer excitons in $MoSe_2$/2D perovskite hybrid heterostructures–the interplay between charge and energy transfer, Nanoscale **14**, 8085 (2022).

[36] W. Li, X. Lu, S. Dubey, L. Devenica, and A. Srivastava, Dipolar interactions between localized interlayer excitons in van der Waals heterostructures, Nature Materials **19**, 624 (2020).

[37] P. Rivera, K. L. Seyler, H. Yu, J. R. Schaibley, J. Yan, D. G. Mandrus, W. Yao, and X. Xu, Valley-polarized exciton dynamics in a 2D semiconductor heterostructure, Science **351**, 688 (2016).

[38] A. R.-P. Montblanch, D. M. Kara, I. Paradisanos, C. M. Purser, M. S. Feuer, E. M. Alexeev, L. Stefan, Y. Qin, M. Blei, G. Wang, A. R. Cadore, P. Latawiec, M. Lončar, S. Tongay, A. C. Ferrari, and M. Atatüre, Confinement of long-lived interlayer excitons in WS_2/WSe_2 heterostructures, Communications Physics **4**, 119 (2021).

[39] L. Butov, A. Shashkin, V. Dolgopolov, K. Campman, and A. Gossard, Magneto-optics of the spatially separated electron and hole layers in GaAs/$Al_xGa_{1-x}As$ coupled quantum wells, Physical Review B **60**, 8753 (1999).

[40] L. A. Jauregui, A. Y. Joe, K. Pistunova, D. S. Wild, A. A. High, Y. Zhou, G. Scuri, K. De Greve, A. Sushko, C.-H. Yu, T. Taniguchi, K. Watanabe, D. J. Needleman, M. D. Lukin, H. Park, and P. Kim, Electrical control of interlayer exciton dynamics in atomically thin heterostructures, Science **366**, 870 (2019).

[41] O. Karni, E. Barré, S. C. Lau, R. Gillen, E. Y. Ma, B. Kim, K. Watanabe, T. Taniguchi, J. Maultzsch, K. Barmak, R. H. Page, and T. F. Heinz, Infrared interlayer exciton emission in MoS_2/WSe_2 heterostructures, Physical Review Letters **123**, 247402 (2019).

[42] P. Nagler, G. Plechinger, M. V. Ballottin, A. Mitioglu, S. Meier, N. Paradiso, C. Strunk, A. Chernikov, P. C. Christianen, C. Schüller, and T. Korn, Interlayer exciton dynamics in a dichalcogenide monolayer heterostructure, 2D Materials **4**, 025112 (2017).

[43] Z. Liu, S. Liu, Y. Chen, J. Hu, H. Wang, Y. Gao, and D. Li, Probing phase distribution at the surface of $(BA)_2(MA)_{n-1}Pb_nI_{3n+1}$ perovskite film via interlayer exciton emission, Advanced Optical Materials **13**, e01819 (2025).

[44] W. Yao, D. Yang, Y. Chen, J. Hu, J. Li, and D. Li, Layer-number engineered momentum-indirect interlayer excitons with large spectral tunability, Nano Letters **22**, 7230 (2022).

[45] B. Laikhtman and R. Rapaport, Exciton correlations in coupled quantum wells and their luminescence blue shift, Physical Review B **80**, 195313 (2009).

[46] K. P. O'Donnell and X. Chen, Temperature dependence of semiconductor band gaps, Applied Physics Letters **58**, 2924 (1991).

[47] S. Tongay, J. Zhou, C. Ataca, K. Lo, T. S. Matthews, J. Li, J. C. Grossman, and J. Wu, Thermally driven crossover from indirect toward direct bandgap in 2D semiconductors: $MoSe_2$ versus MoS_2, Nano Letters **12**, 5576 (2012).

[48] J. S. Ross, S. Wu, H. Yu, N. J. Ghimire, A. M. Jones, G. Aivazian, J. Yan, D. G. Mandrus, D. Xiao, W. Yao, and X. Xu, Electrical control of neutral and charged excitons in a monolayer semiconductor, Nature Communications **4**, 1474 (2013).

[49] J. Huang, T. B. Hoang, and M. H. Mikkelsen, Probing the origin of excitonic states in monolayer WSe_2, Scientific Reports **6**, 22414 (2016).

[50] J. Fu, M. Li, A. Solanki, Q. Xu, Y. Lekina, S. Ramesh, Z. X. Shen, and T. C. Sum, Electronic states modulation by coherent optical phonons in 2D halide perovskites, Advanced Materials **33**, 2006233 (2021).

[51] J. N. Wilson, J. M. Frost, S. K. Wallace, and A. Walsh, Dielectric and ferroic properties of metal halide perovskites, APL Materials **7** (2019).

[52] T. Godde, D. Schmidt, J. Schmutzler, M. Aßmann, J. Debus, F. Withers, E. Alexeev, O. Del Pozo-Zamudio, O. Skrypka, K. Novoselov, M. Bayer, and A. Tartakovskii, Exciton and trion dynamics in atomically thin $MoSe_2$ and WSe_2: Effect of localization, Physical Review B **94**, 165301 (2016).

[53] X.-X. Zhang, Y. You, S. Y. F. Zhao, and T. F. Heinz, Experimental evidence for dark excitons in monolayer WSe_2, Physical Review Letters **115**, 257403 (2015).

[54] H. Shibata, Negative thermal quenching curves in photoluminescence of solids, Japanese Journal of Applied Physics **37**, 550 (1998).

[55] Y. Fang, L. Wang, Q. Sun, T. Lu, Z. Deng, Z. Ma, Y. Jiang, H. Jia, W. Wang, J. Zhou, and H. Chen, Investigation of temperature-dependent photoluminescence in multi-quantum wells, Scientific Reports **5**, 12718 (2015).

[56] Z. Guo, X. Wu, T. Zhu, X. Zhu, and L. Huang, Electron–phonon scattering in atomically thin 2D perovskites, ACS Nano **10**, 9992 (2016).

[57] Y. Chen, H. Wang, and D. Li, Interlayer coupling in two-dimensional perovskite/monolayer transition metal dichalcogenide heterostructures, 2D Materials **12**, 033005 (2025).

[58] X. Li, A. A. Puretzky, X. Sang, S. KC, M. Tian, F. Ceballos, M. Mahjouri-Samani, K. Wang, R. R. Unocic, H. Zhao, G. Duscher, V. R. Cooper, C. M. Rouleau, D. D. Geohegan, and K. Xiao, Suppression of defects and deep levels using isoelectronic tungsten substitution in monolayer $MoSe_2$, Advanced Functional Materials **27**, 1603850 (2017).

[59] T. Y. Jeong, H. Kim, S.-J. Choi, K. Watanabe, T. Taniguchi, K. J. Yee, Y.-S. Kim, and S. Jung, Spectroscopic studies of atomic defects and bandgap renormalization in semiconducting monolayer transition metal dichalcogenides, Nature Communications **10**, 3825 (2019).

[60] I. Levine, D. Menzel, A. Musiienko, R. MacQueen, N. Romano, M. Vasquez-Montoya, E. Unger, C. Mora Perez, A. Forde, A. J. Neukirch, L. Korte, and T. Dittrich, Revisiting sub-band gap emission mechanism in 2D halide perovskites: the role of defect states, Journal of the American Chemical Society **146**, 23437 (2024).

[61] H.-H. Fang, E. K. Tekelenburg, H. Xue, S. Kahmann, L. Chen, S. Adjokatse, G. Brocks, S. Tao, and M. A. Loi, Unraveling the broadband emission in mixed tin-lead layered perovskites, Advanced Optical Materials **11**, 2202038 (2023).

[62] G. Finkelstein, H. Shtrikman, and I. Bar-Joseph, Optical spectroscopy of a two-dimensional electron gas near the metal-insulator transition, Physical Review Letters **74**, 976 (1995).

[63] K. F. Mak, K. He, J. Shan, and T. F. Heinz, Control of valley polarization in monolayer MoS_2 by optical helicity, Nature Nanotechnology **7**, 494 (2012).

[64] S. Kahmann, E. K. Tekelenburg, H. Duim, M. E. Kamminga, and M. A. Loi, Extrinsic nature of the broad photoluminescence in lead iodide-based Ruddlesden–Popper perovskites, Nature Communications **11**, 2344 (2020).

[65] S. Tongay, J. Suh, C. Ataca, W. Fan, A. Luce, J. S. Kang, J. Liu, C. Ko, R. Raghunathanan, J. Zhou, F. Ogletree, J. Li, J. C. Grossman, and J. Wu, Defects activated photoluminescence in two-dimensional semiconductors: interplay between bound, charged and free excitons, Scientific Reports **3**, 2657 (2013).

[66] W. Li, L. Hu, J. Ma, C. Jiang, S. Zhang, Y. Chen, J. Hu, X. Liu, T. Wu, and D. Li, Enhancing self-trapped exciton emission via energy transfer in two-dimensional/quantum dot perovskite

heterostructures, ACS Photonics **9**, 2008 (2022).

[67] F. Pan, J. Li, X. Ma, Y. Nie, B. Liu, and H. Ye, Free and self-trapped exciton emission in perovskite $CsPbBr_3$ microcrystals, RSC Advances **12**, 1035 (2022).

[68] M. D. Smith, A. Jaffe, E. R. Dohner, A. M. Lindenberg, and H. I. Karunadasa, Structural origins of broadband emission from layered Pb–Br hybrid perovskites, Chemical Science **8**, 4497 (2017).

[69] Y. Han, X. Cheng, and B.-B. Cui, Factors influencing self-trapped exciton emission of low-dimensional metal halides, Materials Advances **4**, 355 (2023).

[70] A. Castellanos-Gomez, X. Duan, Z. Fei, H. R. Gutierrez, Y. Huang, X. Huang, J. Quereda, Q. Qian, E. Sutter, and P. Sutter, Van der Waals heterostructures, Nature Reviews Methods Primers **2**, 58 (2022).

[71] H. Li, Y. Qin, B. Shan, Y. Shen, F. Ersan, E. Soignard, C. Ataca, and S. Tongay, Unusual pressure-driven phase transformation and band renormalization in 2D vdW hybrid lead halide perovskites, Advanced Materials **32**, 1907364 (2020).

[72] D. Spirito, M. Barra-Burillo, F. Calavalle, C. L. Manganelli, M. Gobbi, R. Hillenbrand, F. Casanova, L. E. Hueso, and B. Martín-García, Tailoring photoluminescence by strain-engineering in layered perovskite flakes, Nano Letters **22**, 4153 (2022).

[73] E. Blundo, E. Cappelluti, M. Felici, G. Pettinari, and A. Polimeni, Strain-tuning of the electronic, optical, and vibrational properties of two-dimensional crystals, Applied Physics Reviews **8**, 021318 (2021).

[74] T. Yin, B. Liu, J. Yan, Y. Fang, M. Chen, W. K. Chong, S. Jiang, J.-L. Kuo, J. Fang, P. Liang, S. Wei, K. P. Loh, T. C. Sum, T. J. White, and Z. X. Shen, Pressure-engineered structural and optical properties of two-dimensional $(C_4H_9NH_3)_2PbI_4$ perovskite exfoliated nm-thin flakes, Journal of the American Chemical Society **141**, 1235 (2018).

[75] H. M. Hill, A. F. Rigosi, A. Raja, A. Chernikov, C. Roquelet, and T. F. Heinz, Exciton broadening in WS_2/graphene heterostructures, Physical Review B **96**, 205401 (2017).
